# Muscle in Variable Gravity: “I Do Not Know Where I Am, But I Know What to Do”

**DOI:** 10.3389/fphys.2021.714655

**Published:** 2021-08-04

**Authors:** Elena Monti, Janice Waldvogel, Ramona Ritzmann, Kathrin Freyler, Kirsten Albracht, Michael Helm, Niccolò De Cesare, Piero Pavan, Carlo Reggiani, Albert Gollhofer, Marco Vincenzo Narici

**Affiliations:** ^1^Department of Biomedical Science, University of Padova, Padova, Italy; ^2^Department of Sport and Sport Science, University of Freiburg, Freiburg, Germany; ^3^Department of Biomechanics, Rennbahnklinik, Muttenz, Switzerland; ^4^Institute of Movement and Neurosciences, German Sport University Cologne, Cologne, Germany; ^5^Department of Medical Engineering and Technomathematics, Aachen University of Applied Sciences, Aachen, Germany; ^6^Department of Industrial Engineering, University of Padova, Padova, Italy

**Keywords:** parabolic flight, drop jump, hypo-gravity, hyper-gravity, sarcomere operating length

## Abstract

**Purpose:** Fascicle and sarcomere lengths are important predictors of muscle mechanical performance. However, their regulation during stretch-shortening cycle (SSC) activities in usual and challenging conditions is poorly understood. In this study, we aimed to investigate muscle fascicle and sarcomere behavior during drop jumps (a common SSC activity) in conditions of variable gravity.

**Methods:** Fifteen volunteers performed repeated drop jumps in 1 g, hypo-gravity (0 to 1 g), and hyper-gravity (1 to 2 g) during a parabolic flight. Gastrocnemius medialis (GM) electromyographic activity and fascicle length (Lf) were measured at drop-off, ground contact (GC), minimum ankle joint angle (MAJ), and push-off. GM sarcomere number was estimated by dividing Lf, measured by ultrasound at rest, by published data on GM sarcomere length, and measured *in vivo* at the same joint angle. Changes in sarcomere length were estimated by dividing GM Lf in each jump phase by sarcomere number calculated individually. The sarcomere force-generating capacity in each jump phase was estimated from the sarcomere length-tension relationship previously reported in the literature.

**Results:** The results showed that, regardless of the gravity level, GM sarcomeres operated in the ascending portion of their length-tension relationship in all the jump phases. Interestingly, although in hypo-gravity and hyper-gravity during the braking phase (GC-MAJ) GM fascicles and sarcomeres experienced a stretch (as opposed to the quasi-isometric behavior in 1 g), at MAJ they reached similar lengths as in 1 g, allowing sarcomeres to develop about the 70% of their maximum force.

**Conclusion:** The observed fascicle behavior during drop jumping seems useful for anchoring the tendon, enabling storage of elastic energy and its release in the subsequent push-off phase for effectively re-bouncing in all gravity levels, suggesting that an innate neuromuscular wisdom enables to perform SSC movements also in challenging conditions.

## Introduction

Performing tasks, such as running and jumping, requires activation of the agonist and antagonist muscles before (motor unit pre-activation) and during movement performance (Santello and Mcdonagh, 1998). A well-timed and regulated muscle activation elicits a stretch-shortening cycle (SSC) response, naturally occurring in bouncing movements (Ishikawa and Komi, 2004; Taube et al., 2012). By definition, the SSC describes the stretching of a pre-activated muscle-tendon complex immediately followed by a muscle shortening in the concentric push-off phase (Komi, 1984).

Given the importance of SSC actions for human movement, it is not surprising that many studies investigated the biomechanics of this phenomenon; in particular, drop jumps (DJs) represent a good paradigm to study muscle fascicle and tendon behavior in ballistic movements involving the SSC.

Within a DJ, three main phases [pre-activation, braking, and push-off (PO; Komi, 2000)] have been recognized and extensively studied in common and challenging conditions, such as changes in load, falling height, or simulated hypo-gravity (Avela et al., 1994; Arampatzis et al., 2001; Fukashiro et al., 2005; Ishikawa et al., 2005; Sousa et al., 2007; Ritzmann et al., 2016; Helm et al., 2020).

These studies show that the timing and amount of triceps-surae muscle-tendon unit pre-activation in DJs are differentially regulated based on the load applied to the muscle, being optimal in normal “Earth” gravity conditions (Avela et al., 1994), but decreased in simulated hypo-gravity, hyper-gravity (Avela et al., 1994; Ritzmann et al., 2016), or unknown conditions (i.e., unknown falling heights; Helm et al., 2020). Some authors indicated that, when falling from heights different from the optimal one [defined as the drop height giving a maximum DJ performance indicated as peak ground reaction force (GRF) or jump high], electromyographic (EMG) activity of the plantar flexors increases from lower than optimal to higher than optimal heights (Ishikawa and Komi, 2004; Sousa et al., 2007).

These findings highlight the ability of the central nervous system to regulate the timing and amount of pre-activation according to different jumping conditions, thus regulating muscle fascicle length, tendon and joint stiffness as well as position, in order to safely land on the ground and quickly re-bounce.

Similarly, to pre-activation, also in the braking phase, the plantar flexors are differentially regulated. In optimal height (i.e., load) jumping conditions, gastrocnemius medialis (GM) fascicles shorten at early ground contact (possibly due to the intervention of the stretch reflex; Gollhofer et al., 1992) and behave quasi-isometrically in the late braking phase, enabling tendon elongation, and storage of elastic energy (Gollhofer et al., 1992; Fukashiro et al., 2005; Sousa et al., 2007). When increasing the falling height (augmenting the impact GRF), the quasi-isometric behavior of fascicles disappears, and fast fascicle lengthening occurs (Ishikawa et al., 2005; Sousa et al., 2007).

In the third and last PO phase, fascicles shorten and the tendon releases the elastic energy previously stored. Bobbert et al. (1987) reported no influence of jumping height on the work done and on the net vertical impulse assessed during PO; this observation suggests that, despite an optimal DJ performance might be achieved only in specific conditions (falling heights, loads), the central nervous system seems to be able to regulate muscle behavior in order to effectively perform the required task also in challenging situations.

Although the regulation of triceps-surae muscle-tendon unit in DJs has been extensively investigated, very few studies focused on sarcomeres behavior during the performance of this SSC movement (Kurokawa et al., 2003; Fukashiro et al., 2005, 2006). Sarcomeres represent muscle contractile units and are known to express different amounts of force depending on their length (Gordon et al., 1966; Walker and Schrodt, 1974); thus, understanding the time course of their responses during DJs is fundamental to gain further insights into muscle force-generating capacity. *In vivo* measurement of sarcomere length in humans has been so far been performed only in static positions and under highly controlled experimental conditions (Llewellyn et al., 2008; Sanchez et al., 2015). Instead, human sarcomere length estimation (achieved by dividing GM measured fascicle length for a fixed sarcomere number) in dynamic contractions provided an indirect measure of sarcomere operating range during squat jump, countermovement jump, and DJ (Fukashiro et al., 2005, 2006; Kurokawa et al., 2003). The results of these studies showed that sarcomeres operate in the ascending limb of their length-tension (L-T) relationship in all types of jumps, and particularly so in DJ.

However, most of the available observations on sarcomere and muscle fascicle behavior were made in condition of constant gravity. Thus, in order to understand how sarcomere and muscle fascicle length are regulated in variable gravity conditions, we performed experiments in a parabolic flight, involving variable gravity levels, ranging from about zero-g to about double the Earth’s gravity (1 g; Waldvogel et al., 2021).

Specifically, the aims of the present study were as follows:

1. To investigate the ability of the neuromuscular system in regulating fascicle length in response to conditions of variable gravity.
2. To estimate sarcomere operative length in the different DJ phases, in order to calculate its theoretical force production and its possible modulation in conditions of variable gravity.

We hypothesized that muscle fascicles would be differentially regulated in different gravity conditions compared to 1 g, particularly in anticipation of landing and re-bouncing in unknown gravity levels. In addition, we hypothesized that sarcomeres would operate in the upper part of the ascending limb of their L-T relationship, possibly lengthening during the braking phase (especially in hyper-gravity) while operating quasi-isometrically in 1 g.

## Materials and Methods

### Participants

Seventeen healthy participants (7 females and 10 males, height 175 ± 9 cm, body mass 72 ± 12 kg, age 31 ± 5 years) were recruited for the study (Waldvogel et al., 2021). Among them, two males were excluded from the analysis reported in this study due to low ultrasound data quality. All participants gave written informed consent to the experimental procedures. All procedures were (i) in accordance with the latest revised version of the Declaration of Helsinki, (ii) approved by the French authorities (DEMEB of the AFSSAPS) responsible for the protection of subjects participating in biomedical research, and (iii) approved by the Ethics Committee of the University of Freiburg (430/17).

Each participant underwent a visit with a physician specialized in space medicine in order to assess his/her anamnesis, blood pressure, and electrocardiogram and to confirm the absence of any possible conditions that may have precluded him/her from flying. Exclusion criteria were pregnancy, any sickness, neurologic or orthopedic injuries, vestibular or proprioceptive dysfunction, fear of flying, previous surgeries on the left or right leg, neuro-degenerative diseases, or single events associated with neural dysfunctions. Inclusion criteria were a reliable reactive jump pattern and experience in parabolic flights (≥one flight).

### Parabolic Flight

**Figure 1:**
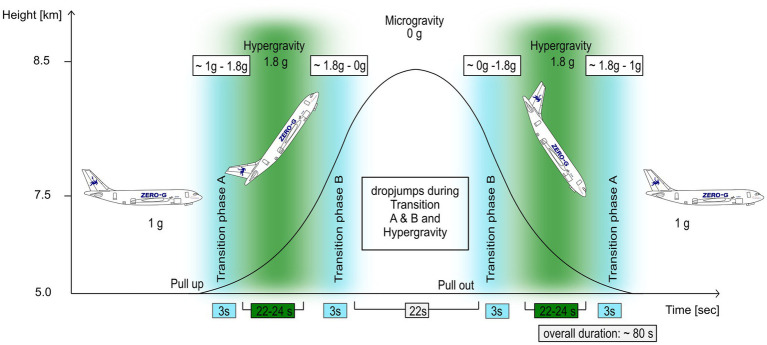
The parabolic flight maneuver and the corresponding gravity levels: Each parabola starts with the airplane flying in “steady flight” condition (Earth’s gravity), followed by a hyper-gravity phase. When the aircraft reaches an angle of 50°, a free fall condition (zero gravity) is faced, after which a new hyper-gravity phase is experienced. The parabola ends with a new “steady-flight” phase. Jumps were performed during the hyper-gravity (green) and the transition phases A and B (blue); reference jumps were performed during 1 g. Adapted from Waldvogel et al. (2021).

A parabolic flight consists of repeated parabolic trajectories that provide short-duration periods of free fall (zero gravity) alternating with high-g pull-up or recovery phases (Shelhamer, 2016; Figure 1). Specifically, each parabola starts with the airplane flying in the so-called “steady flight” condition (Earth’s gravity). As the parabola begins, a hyper-gravity phase is entered, reaching gravity values almost doubled than the Earth’s one. When the aircraft reaches an angle of 50°, a period of about 20–30 s of free fall (zero gravity) is experienced, after which the aircraft enters the descending limb of the parabola and a new hyper-gravity phase occurs. The parabola ends with a new “steady-flight” phase.

The experiments were performed on nine different parabolic flights promoted by ESA (69th and 70th campaign) and DLR (33rd campaign) and conducted by Novespace, in Merignac (BOD, France); each flight involved thirty-one parabolas.

### Data Collection Protocol

Data collection was performed as described in Waldvogel et al. (2021).

Briefly, before flying, participants performed a 4-week training consisting of repetitive sets of drop jumps in order (i) to avoid learning effect during the experiment and (ii) to be familiarized and optimize the jump performance.

During the pre-flight measurements, each participant was required to first stand on a 25-cm height platform (10 s bipedal standing and 10 s with the right foot fully relaxed) and then perform reference DJs in the Earth’s gravity condition. In two participants, reference DJs were performed during steady flight (1 g condition).

Each participant took part in one of the nine flights included in the study. Two participants per flight were tested.

During each parabola, participants performed six DJs from the same pre-test 25-cm height platform in different gravitational conditions. A trained operator informed the participant on the precise jump timing in pre-determined time points during the parabola. Specifically, jumps were performed in the following order: DJ in the first transition phase A, DJ in the first hyper-gravity phase, DJ in the first transition phase B, DJ in the second transition phase B, DJ in the second hyper-gravity phase, and DJ in the second transition phase A (Figure 1).

Each participant was tested during 15 parabolas. Fatigue was avoided by the rest periods in between the parabolas and sets of DJs, respectively (~2 min).

**Figure 2:**
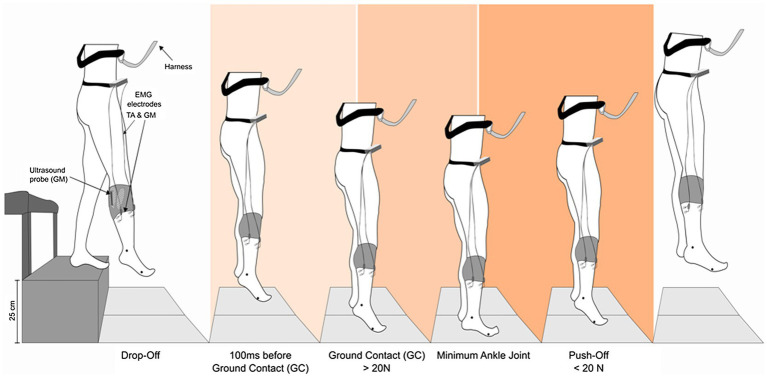
Schematic overview of the experimental setup. Subjects performed drop jumps from a rack with a drop height of 25 cm to separated AMTI force plates. Additionally, they were safely attached to a harness during the whole flight. Electromyographic activity (EMG) of the gastrocnemius medialis (GM) and tibialis anterior (TA) muscles was recorded using Ag/CL electrodes. For monitoring the GM fascicle behavior, a linear ultrasound probe was placed on the muscle belly and secured with a unique device in order to avoid probe’s movements against the skin. 2D kinematic markers were placed on the malleolus and the fifth metatarsal bone.

GM and tibialis anterior (TA) EMG and GM fascicles behavior were examined, as this muscle has been shown to be highly active and crucial for ankle joint stiffness and power generation when bouncing in different gravity levels (Ritzmann et al., 2016). The experimental setting is shown in Figure 2.

### Jump Analysis and Gravity Clustering

Within each DJ, five different time points were considered for the analysis: (i) the drop-off (DO) consisting in the time point 12 ms before the participant started to fall from the platform – that is the muscle condition at the beginning of the DJ, (ii) 100 ms before ground contact (100 ms before GC) – during pre-activation, (iii) ground contact (GC) being the end of the pre-activation and the beginning of the braking phase, (iv) the minimum ankle joint angle (MAJ), consisting in the end of the braking phase, and (v) the push-off (PO), representing the last frame before the ground was left and the flight phase was entered (Figure 2).

The jump was recorded by a high-speed camera (Basler ace acA1920, Basler AG., Ahrensburg, sampling frequency of 100 Hz), placed at a distance of 1 m from the force platform and perpendicular to the sagittal plane (in accordance with Gambelli et al., 2016), in order to detect the different jump phases. A trigger was used to synchronize the jump beginning in the camera video, as well as the electromyographic, ultrasonography, and force recordings (see the following Methods section).

The detection and division of DJ in five different time points (DO, 100 ms before GC, GC, MAJ, and PO) were performed as follows: for DO, the ultrasound frame immediately before starting the fall (individuated by a trained operator from visual inspection of the DJ video recording) was chosen; for GC and PO, the ultrasound frame corresponding to the time point when the GRF would exceed or fall below the threshold of 20 N; for MAJ, the ankle angle from the kinematic analysis was utilized; 100 ms before GC was calculated as 100 ms before GC occurred.

For each jump, acceleration data (x, y, and z components) were acquired with an accelerometer, placed at the experimental rack, to detect the instantaneous gravity force. After calculating the average gravity of each DJ (see below), this was assigned to one of nine gravity clusters ranging from 0 to 2 g, considering 1 g as the Earth’s gravity, which are reported in Table 1.

**Table 1:** Gravity levels identified by obtaining the gravity for three discrete DJ time points (150 ms before ground contact, instant of GC, and instant of PO) and two time intervals (from GC until PO and from 150 ms before GC until PO) – see Methods.

Gravity level	Gravity mean ± SD
0–0.25 g	0.19 ± 0.06
0.25–0.5 g	0.39 ± 0.08
0.5–0.75 g	0.62 ± 0.08
0.75–1 g	0.91 ± 0.07
1 g	1.00 ± 0.05
1–1.25 g	1.11 ± 0.08
1.25–1.5 g	1.41 ± 0.07
1.5–1.75 g	1.64 ± 0.08
1.75–2 g	1.81 ± 0.05

*Mean gravity and standard deviation for each gravity level is reported*.

To cluster the gravity levels, the exact gravity level for each DJ was defined for three discrete time points (150 ms before ground contact, instant of GC, and instant of PO) and two time intervals (from GC until PO and from 150 ms before GC until PO) based on the acceleration along the axis perpendicular to the floor of the plane. Trials were excluded if vibrations or turbulences occurred. Inconsistent gravity levels characterized by high deviations during the DJs (above 0.3 g, thus above the 0.25 gravity range of each cluster) were excluded from further analysis. This ensured that only conditions within upper or lower predefined boundaries as well as smooth trajectories without vibrations were included. Table 1 reports means and standard deviations observed within the wider time interval considered (from 150 ms before GC until PO) for the nine gravity clusters.

### Force Measurements

**Figure 3:**
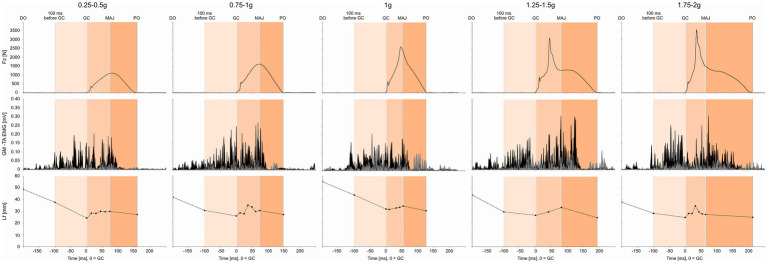
Ground reaction forces (GRF), tibialis anterior (TA, gray), and gastrocnemius medialis (GM, black) EMG and fascicle length (Lf) traces for a representative subject in five of the nine gravity levels analyzed (0.25–0.5 g, 0.75–1 g, 1 g, 1.25–1.5 g, and 1.75–2 g).

Ground reaction forces for the left and right legs were recorded with a separated AMTI force plate (OR6-6, AMTI, Watertown, United States) with a sampling frequency of 2 kHz (Figures 2, 3).

### Kinematics Measurements

The 2D kinematics of the right limb was recorded with the same high-speed camera used to detect the different jump phases. Markers were taped on the participants’ skin on the lateral epicondyle, lateral malleolus, and fifth metatarsal (Gambelli et al., 2016). SIMI Motion software (SIMI Reality Motions System GmbH, 85716 Unterschleißheim, Germany) was used for the kinematic recording and analyses.

### EMG Measurements and Analysis

#### EMG Recording

Bipolar Ag/AgCl surface electrodes (Ambu Blue Sensor P, Ballerup, Denmark, diameter 9 mm, center-to-center distance 34 mm) were placed over the right leg tibialis anterior (TA) and GM muscles bellies. The longitudinal axes of the electrodes were in line with the presumed direction of the underlying muscle fibers. The reference electrode was placed on the lower part of the tibia. Before placing the electrodes, the skin was gently shaved, abraded, and disinfected in order to reduce the inter-electrode resistance below 5 kΩ. Procedures were performed according to SENIAM (Hermens et al., 2000). The EMG signals were transmitted *via* shielded cables to the amplifier (band-pass filter 20 Hz to 1 kHz, 200× amplified) and recorded with 2 kHz (A/D conversion *via* a National Instruments PCI-6229 DAQ-card, 16bit resolution). Before flying, GM and TA maximum voluntary contraction (MVC) were acquired. The highest EMG values were used to normalize data for each volunteer (EMG maximum peak ± 25 ms). The MVCs were executed according to Wiley and Damiano (1998) and Roelants et al. (2006), performed isometrically (at joint level) against an unmovable resistance, and held for 3 s with 1 min in between. A trained operator supervised MVC performance, in order to strictly control and standardize body position and MVC performance, while providing strong verbal encouragement. The EMG electrodes were placed before MVC testing and afterward maintained during both reference measurements in 1 g and experimental data collection during the parabolic flights.

#### EMG Analysis

For each of the five jump time points (DO, 100 ms before GC, GC, MAJ, and PO, see Figure 2), EMG raw signal of TA and GM was calculated from the correspondent ±25 ms time interval. The EMG activity was then rectified, averaged, integrated (iEMG), and time normalized (to 1 s). Finally, all the values were normalized for the EMG at MVC of the corresponding muscle. GM/TA EMG ratio was then calculated.

### Ultrasound Recordings and Analysis

#### Ultrasonography Recordings

Muscle architecture variations during jumps were recorded using B-mode ultrasound at a frame frequency of 82 Hz. The transducer was positioned over the GM muscle belly to visualize fascicles and aponeuroses according to Werkhausen et al. (2018). The transducer (96-element, 6 cm linear-array probe, B-mode, frequency of 7 MHz, imaging depth of 50 mm and width of 60 mm) was securely fastened to the skin with adhesive tape at the interface of plastic frame, built by a 3D printer, to avoid probe movement above the skin. The ultrasound data were recorded with a Telemed Echo Wave II – Vers. 3.6.2 (Telemed Ltd., Lithuania) system.

#### Ultrasound Image Analysis

**Figure 4:**
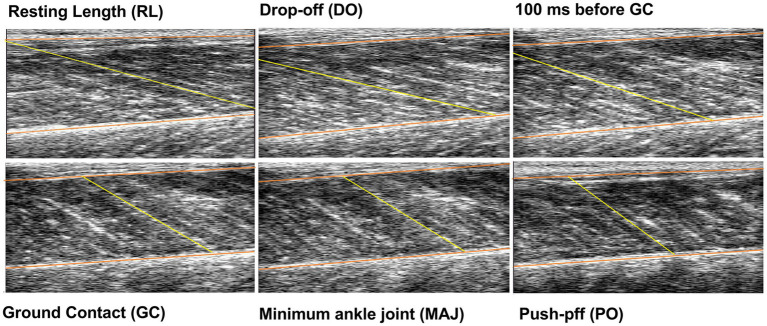
Ultrasound images extrapolated from the video recorded during a drop jump (DJ) from a representative subject. The chosen jump time points are represented: resting length (RL; the participant being bipedally standing on the platform with 0° of ankle and knee flexion), drop-off (DO; 12 ms before starting to fall), 100 ms before ground contact (GC; during pre-activation), GC (beginning of the braking phase), minimum ankle joint angle (MAJ; end of the braking phase), and push-off (PO; representing the last frame before the ground is left and the flight phase is entered). One of the three analyzed fascicles and the superficial and deep aponeuroses have been superimposed to the images in yellow and orange, respectively.

Ultrasound videos were converted in QTFF to be analyzed in a custom-made semi-automatic software (MATLAB). The image frame corresponding to the five different jump time points (DO, 100 ms before GC, GC, MAJ, and PO) and the image frames between GC and MAJ were selected for analysis. GM fascicle length (Lf, the length of the inclined GM fibers calculated as the intersection of the single fascicle with the upper and lower aponeurosis) was calculated as the average of three fascicles per image (see section below). Individual DO Lf (measured in each gravity level) was used to normalize Lf changes throughout the jump time points. Images obtained from a representative participant (resting length, DO, 100 ms before GC, GC, MAJ, and PO) are displayed in Figure 4.

### Ultrasound Image Analysis Software Implementation

A custom software was developed in MATLAB R2019a (MathWorks, Natick, United States). The software works as follows. Single video frames are extracted one by one, allowing the operator to choose only the frames of interest in the video sequence using the software command line. A threshold procedure and several morphological structure operations are carried out to automatically determine the lower and upper muscle aponeurosis. Morphological structure operation consists of using a flat element, which in this case is a bi-dimensional binary valued neighborhood, to perform a pixel-by-pixel segmentation and noise canceling on the images in order to recognize the aponeurosis. Afterward, the muscle fibers direction is manually identified by drawing three lines using the graphical interface. The algorithm returns the segmented aponeurosis, the average and standard deviation of the angle between the three identified fibers and the horizontal line, the pennation angle which is the angle between the inferior aponeurosis and the identified fibers, the average and standard deviation of the orthogonal distance between the two aponeuroses, and the average and the standard deviation of the length of the three identified fibers between the aponeuroses. This operation is repeated for each frame initially selected.

Prior to the analysis, the aforementioned software had been validated by analyzing the same jumps in one subject both with the new MATLAB custom-made algorithm and manually with the conventionally used ImageJ free software.^1^ Intraclass correlation coefficient between the two analysis methods was 0.83 [rated as good following the criteria by Koo and Li (2016)].

### Sarcomere Operating Length Calculation

An estimation of the sarcomere number in the GM of each subject was made by dividing the individual fascicle length while standing on the platform (i.e., 0° knee and ankle flexion, corresponding to an angle of 110° at the ankle; see Narici et al., 1996) for the sarcomere resting length of 3.09 μm, measured *in vivo* in the same position by Sanchez et al. (2015).

Thus, in each subject, the fascicle length in the different jump time points was divided by the sarcomere number calculated in order to obtain the average sarcomere operating length in that particular jump time point.

Finally, the obtained sarcomere operative length was superimposed to the length-tension (L-T) relationship described by Walker and Schrodt (1974).

### Statistical Analysis

Normality of the independent variables (GM Lf; GM and TA EMG) was assessed with the following tests: Q-Q plot, skewness and kurtosis calculation, and Shapiro–Wilk normality tests. GM Lf data and ankle joint data were normally distributed. GM Lf data and ankle joint data passed all the normality tests. EMG data, conversely, were not distributed normally. A correction using the logarithm function [Log(x)] was used, and normality was assessed again as previously described.

Since the transformed EMG data passed normality tests, parametric statistics was applied.

Since, in some gravity levels, few subjects were not able to effectively perform DJs (5 subjects for 0–0.25 g, 2 subjects for 1–1.25 g, 2 subjects for 1.5–1.75 g, and 2 subjects for 1.75–2 g) due to turbulences or technical difficulties, data were analyzed by fitting a mixed model instead of two-way repeated measures ANOVA, as this model can deal with random-reason missing values.

Significance was tested (i) among each gravity level between both the different DJ time points (versus the previous frame) and versus baseline (DO) and (ii) at the same DJ time point, comparing each gravity level to 1 g. When the mixed model analysis revealed significant differences, Sidak *post-hoc* test was performed to assess the differences, within a single gravity level, between DO and the following time points and between each time point and the following one, and Tukey *post-hoc* test was used to assess the differences among the gravity levels.

Significance level was set to values of *p* < 0.05.

To test whether the ankle joint at DO could influence the GM Lf, linear relationships between the individual values were calculated using the Pearson’s product–moment correlation coefficient (*r*). The level of significance was set to values of *p* < 0.05.

GraphPad Prism software (version 7.0; GraphPad software Inc., San Diego, CA) was used to perform all statistical and *post-hoc* analysis.

## Results

The time course of the GRF, GM and TA EMG, and GM Lf for a typical participant is shown in Figure 3.

### Ground Reaction Forces

**Figure 5:**
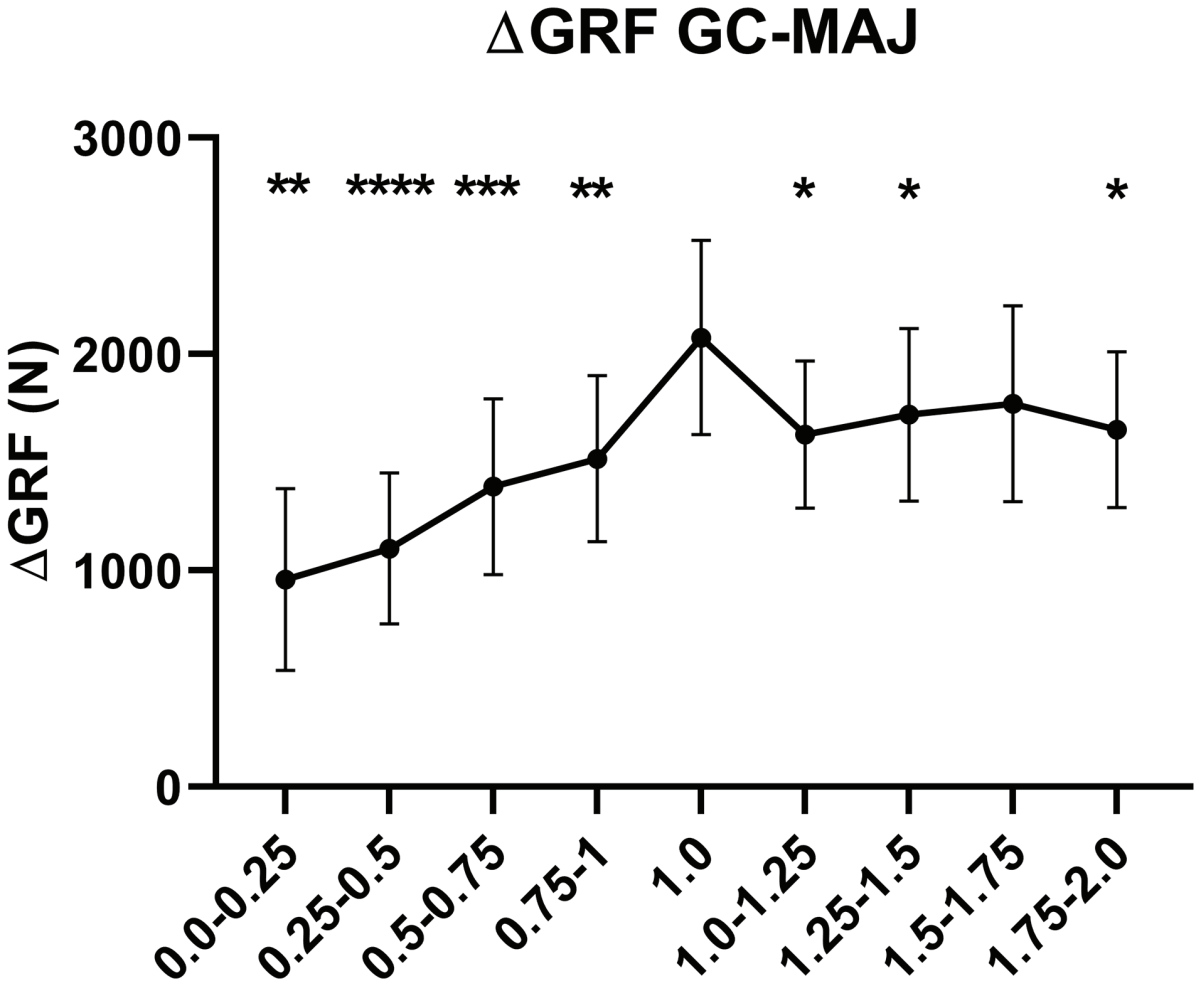
Delta difference in peak Z GRF between ground contact (GC) and MAJ in all the hypo-gravity levels (0–0.25 g, 0.25–0.5 g, 0.5–0.75 g, and 0.75–1 g), hyper-gravity levels (1–1.25 g, 1.25–1.5 g, 1.5–1.75 g, and 1.75–2 g), and 1 g. Results shown as mean ± SD of three jumps per gravity level per subject. ^*^p < 0.05 vs. 1 g; ^**^p < 0.01 vs. 1 g; ^***^p < 0.001 vs. 1 g; ^****^p < 0.0001 vs. 1 g.

Ground reaction force variations between GC and MAJ are shown in Figure 5. Data are reported as the difference between the instantaneous peak Z GRF at GC-MAJ.

The delta in peak Z GRF from GC-MAJ was significantly lower in all the gravity levels below and above 1 g (excluding 1.5–1.75 g) if compared to 1 g, ranging from −56.5% (*p* = 0.004) in 0–0.25 g to −16.1% (*p* = 0.035) in 1.25–1.5 g.

### Time Course of the Changes in GM Lf During DJs Performed in 1 g, Hypo-Gravity and Hyper-Gravity

**Figure 6:**
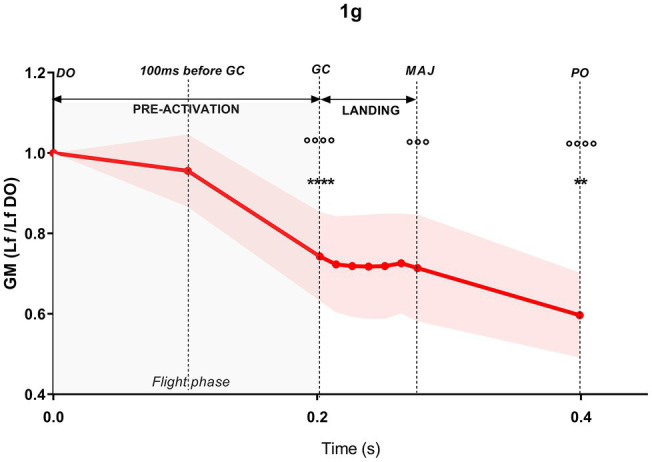
Time course of the gastrocnemius medialis (GM) fascicle length (Lf) variations during drop jumps in 1 g. Values are normalized to drop-off (DO) Lf. Results shown as mean ± SD of maximum three jumps per gravity level per subject. ^**^p < 0.01 vs. previous jump time point; ^****^p < 0.0001 vs. previous jump time point; ^°°^*p* < 0.001 vs. DO; ^°°°°^*p* < 0.0001 vs. DO.

**Figure 7:**
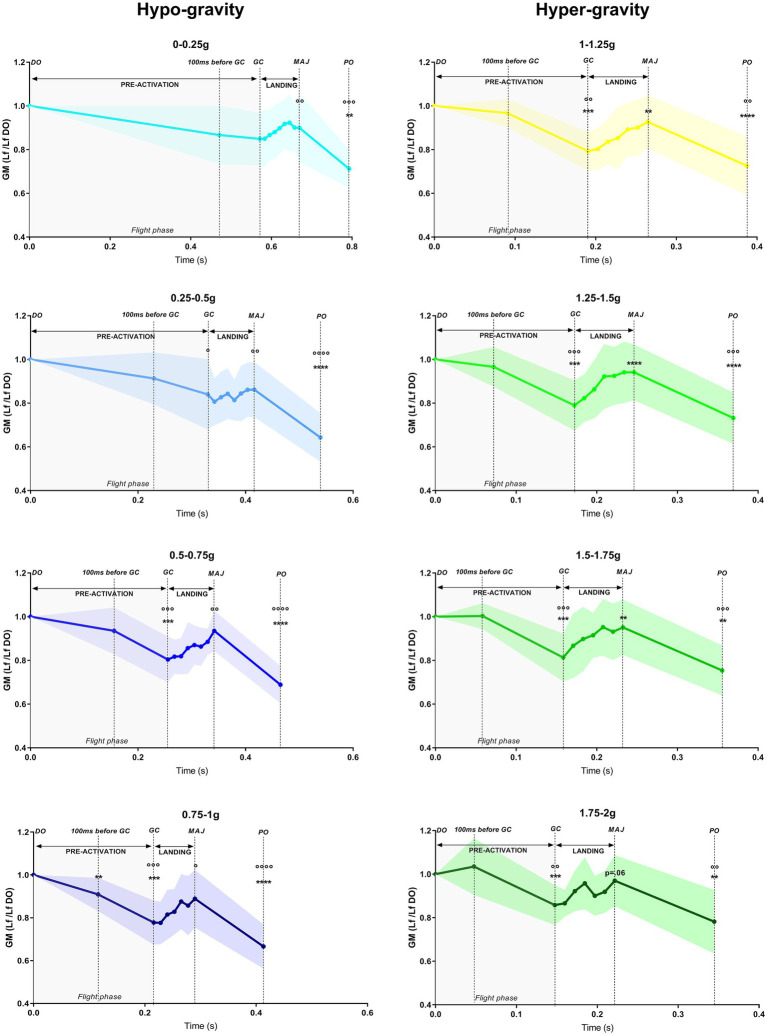
Time course of the gastrocnemius medialis (GM) fascicle length (Lf) variations during drop jumps in hypo-gravity and hyper-gravity. Values are normalized to drop-off (DO) Lf. Results shown as mean ± SD of maximum three jumps per gravity level per subject. ^**^p < 0.01 vs. previous jump time point; ^***^p<0.001 vs. previous jump time point; ^°^*p* < 0.05 vs. DO; ^°°^*p* < 0.01 vs. DO; ^°°°^*p* < 0.001 vs. DO; ^°°°°^*p* < 0.0001 vs. DO.

The time course of the GM Lf changes and the associated muscular activity during DJs per each gravity level is shown in Table 2; Figures 6, 7.

**Table 2:** GM/TA EMG ratio during the five different jump time points in all the nine gravity levels.

	JO	100 ms before GC	GC	MAJ	PO
0–0.25 g	2.047 ± 1.958	1.221 ± 1.077	1.707 ± 0.889	5.069 ± 3.968	0.176 ± 0.172
Log(0–0.25 g)	0.422 ± 0.233	0.304 ± 0.204	0.413 ± 0.137	0.681 ± 0.351	0.067 ± 0.059^*^^,^^°^
0.25–0.5 g	1.945 ± 1.771	1.364 ± 1.018	2.179 ± 1.700	4.846 ± 3.779	0.490 ± 0.650
Log(0.25–0.5 g)	0.401 ± 0.251	0.341 ± 0.170	0.454 ± 0.206	0.678 ± 0.300^*^^,^^°^	0.145 ± 0.150^**^
0.5–0.75 g	2.683 ± 2.540	2.871 ± 3.278	2.414 ± 2.447	5.785 ± 4.675	0.533 ± 0.754
Log(0.5–0.75 g)	0.473 ± 0.280	0.471 ± 0.142	0.457 ± 0.183	0.700 ± 0.220^**^^,^^°^	0.146 ± 0.176^****^^,^^°^
0.75–1 g	2.587 ± 2.778	2.127 ± 1.225	2.117 ± 1.377	4.659 ± 3.031	0.535 ± 0.824
Log(0.75–1 g)	0.465 ± 0.285	0.463 ± 0.144	0.449 ± 0.186	0.695 ± 0.222^***^	0.129 ± 0.181^****^^,^^°°^
1 g	1.224 ± 1.430	2.984 ± 2.563	4.511 ± 4.272	5.715 ± 4.475	0.533 ± 0.663
Log(1 g)	0.285 ± 0.227	0.538 ± 0.229	0.659 ± 0.254^°^	0.752 ± 0.262^°°^	0.155 ± 0.157^****^
1–1.25 g	4.536 ± 2.738	3.242 ± 3.538	2.205 ± 1.785	5.344 ± 4.245	0.517 ± 0.973
Log(1–1.25 g)	0.695 ± 0.217	0.543 ± 0.257	0.460 ± 0.196	0.723 ± 0.272^*^	0.137 ± 0.175^***^^,^^°°°^
1.25–1.5 g	5.049 ± 3.605	2.944 ± 2.260	1.928 ± 1.923	5.871 ± 5.215	0.776 ± 1.446
Log(1.25–1.5 g)	0.710 ± 0.259	0.548 ± 0.200	0.404 ± 0.224^*^^,^^°°^	0.732 ± 0.308^**^	0.173 ± 0.232^***^^,^^°°°^
1.5–1.75 g	3.889 ± 3.522	3.329 ± 3.058	2.345 ± 3.226	4.820 ± 3.688	0.269 ± 0.335
Log(1.5–1.75 g)	0.605 ± 0.271	0.565 ± 0.245	0.427 ± 0.261	0.703 ± 0.231^**^	0.093 ± 0.090^***^^,^^°°°^
1.75–2 g	3.336 ± 2.763	3.938 ± 4.104	2.151 ± 1.732	4.729 ± 4.101	0.198 ± 0.115
Log(1.75–2 g)	0.557 ± 0.280	0.586 ± 0.303	0.443 ± 0.226	0.652 ± 0.329	0.077 ± 0.041^***^^,^^°°°^

*Values are shown as grand means of three jumps per gravity level per subject ± SD. Raw data (gray rows) and transformed data (white rows) are reported*.

* *p < 0.05 vs. previous jump time point;*

** *p < 0.01 vs. previous jump time point;*

*** *p < 0.001 vs. previous jump time point;*

**** *p < 0.0001 vs. previous jump time point;*

° *p < 0.05 vs. JO;*

°° *p < 0.01 vs. JO;*

°°° *p < 0.001 vs. JO*.

In order to understand whether there were gravity-dependent differences in the jump patterns, a comparison between the different jump time points (DO, 100 ms before GC, GC, MAJ, and PO) within each gravity level was performed. Mixed-effect analysis revealed significant jump time point, gravity, and jump time point × gravity interaction for both GM Lf and GM/TA EMG variables.

#### Resting Length and Drop-Off Length

The mean GM Lf while standing on the platform (bipedal standing, 0° of knee and ankle flexion) was 57.4 mm. In this position, the ankle angle is known to be about 110° (Narici et al., 1996).

Since subjects prepared for the jump with their right leg fully extended (from which GM Lf and EMG were measured) and right hip slightly flexed (as shown in Figure 2), all the results are presented as normalized for the DO length (the GM Lf when the subject was preparing for the jump, 12 ms before the beginning of the free fall). In this reference position, the ankle angle was 130° in 1 g and close to 140° in all the other gravity levels (i.e., much more plantar-flexed); thus, GM Lf at DO was always shorter than GM Lf while standing. GM Lf at DO was chosen in order to describe GM muscle fascicles behavior from the beginning to the end of the jump, taking into account possible gravity effects on the muscle before starting the jump.

#### Pre-activation Phase

During the whole pre-activation (DO to GC), GM Lf significantly shortened in all the gravity levels with the exception of 0–0.25 g, ranging from −15.2% in 0.25–0.5 g (*p* = 0.018) to −25.9% in 1 g (*p* < 0.001).

This was accompanied by increases of similar magnitude in GM and TA EMG activity in all the gravity levels [as indirectly deducible observing Figure 3, from a representative subject and as reported in Waldvogel et al. (2021)], so that the ratio GM/TA EMG did not show significant changes (Table 2); the only exceptions were 1 g (+18 folds, *p* = 0.031) and 1.25–1.5 g (−55.2%, *p* = 0.005). A trend for a reduction in GM/TA EMG ratio was also observed in 1–1.25 g (−41.3%, *p* = 0.068). As GM EMG increases, a shortening in Lf is expected; as TA EMG increases, the higher co-activation is expected to stiffen and fix the ankle joint. Thus, by reporting the variations in GM/TA EMG, it is possible to understand how the muscle (Lf) and ankle joints are differentially regulated during the DJ time points.

More in detail, at 100 ms before GC, GM Lf (although being shorter) was not significantly different from DO in any gravity level with the exception of 0.75–1 g (−10.4%, *p* = 0.004), while its activity, together with that of TA, was increased. GM/TA EMG did not change in any gravity level.

From 100 ms before GC to the end of the pre-activation phase (GC), a further Lf shortening was found in all the gravity levels except in 0–0.25 g and 0.25–0.5 g, ranging from about −14% in both 0.5–0.75 and 0.75–1 g (*p* < 0.001) to −23.1% in 1 g (*p* < 0.001). GM and TA EMG kept increasing; thus, no changes in the GM/TA EMG ratio were found in this jump time point when compared to the previous one.

#### Braking Phase

During the braking phase (GC to MAJ), a lengthening of the GM fascicles was observed both in hypo- and the hyper-gravity. The increase in Lf was significant only for the hyper-gravity levels, ranging from +16% in 1–1.25 g (*p* = 0.001) to +21.4% in 1.25–1.5 g (*p* < 0.001). In 1.75–2 g, a strong trend for lengthening was observed (+11.5%, *p* = 0.062). In contrast, in 1 g, GM Lf slightly decreased (−5.2%, n.s.). Once again, GM EMG showed a significant increment in all gravity levels, while TA EMG did not increase to the same extent. Therefore, GM/TA EMG ratio was higher in several gravity levels, excluding 0–0.25 g, 1 g and 1.75–2 g and indicating a differentially regulated GM Lf and ankle joint angle. Among the differentially regulated gravities, GM/TA EMG ratio increases ranged from +1.5-fold (0.75–1 g, *p* < 0.001) to +2.8-fold (1.25–1.5 g, *p* = 0.003).

#### Push-Off Phase

From MAJ to the last PO phase, GM Lf shortened significantly in all gravity levels. Fascicle total shortening ranged from −25.3% in 1.75–2 g (*p* = 0.002) to −41.1% in 1 g (*p* < 0.001) compared to DO length. GM EMG and the ratio between GM/TA dropped significantly for all the gravity levels by 80–90% on average from the previous frame (MAJ).

### Differences in the Time Course of Muscular Behavior During DJs Performed in 1 g, Hypo-Gravity, and Hyper-Gravity

In order to understand whether gravity affected GM Lf and activity, we compared GM fascicle length and muscle activation in the same jump time point among different gravity levels.

**Figure 8:**
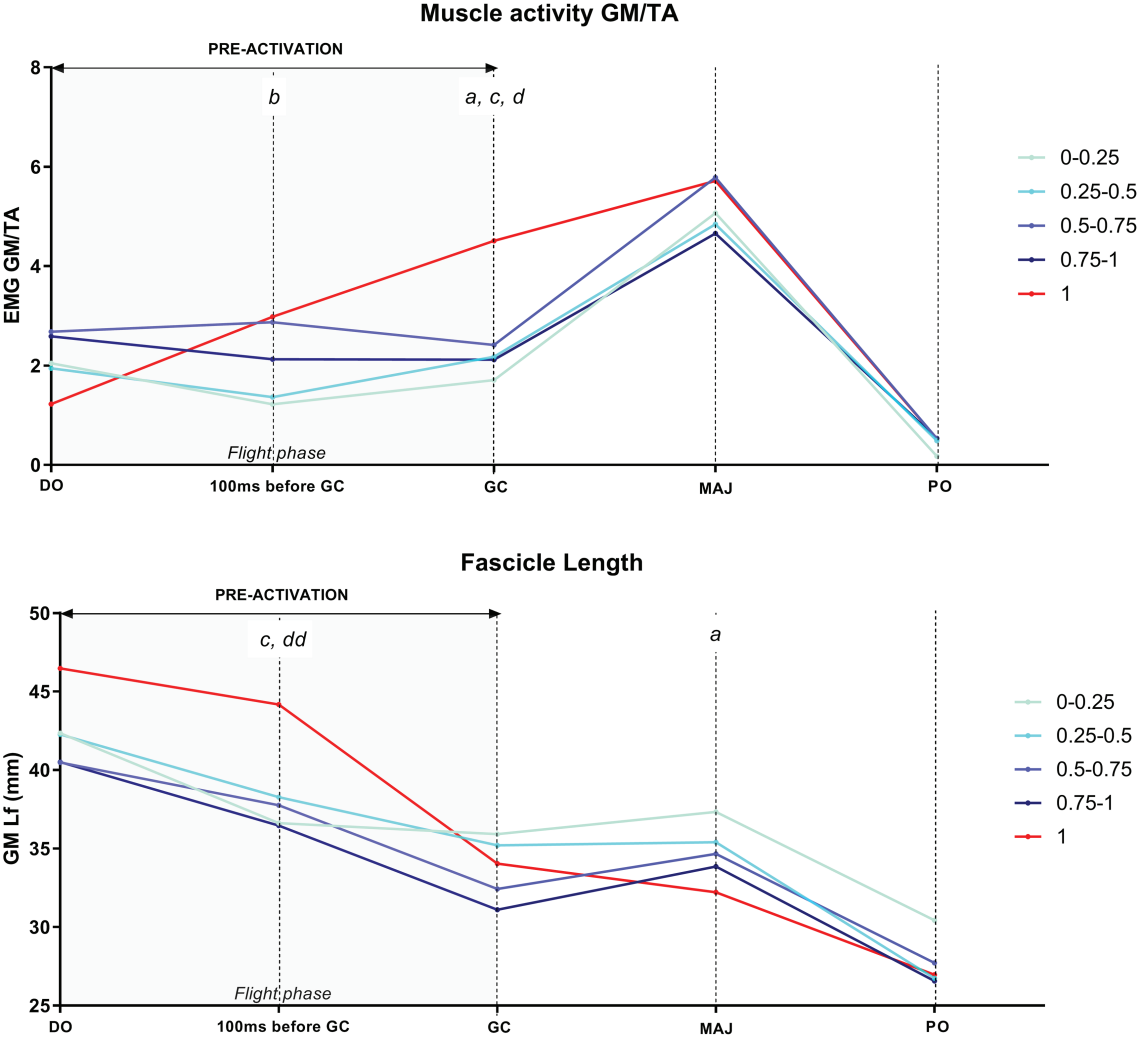
Time course of the gastrocnemius medialis/tibialis anterior (GM/TA) EMG and fascicle length (Lf) during the five drop jump time points – drop-off (DO), 100 ms before ground contact (GC), GC, MAJ, push-off (PO) – in hypo-gravity (0–0.25 g, 0.25–0.5 g, 0.5–0.75 g, and 0.75–1 g) and 1 g. Results shown as grand means of maximum three jumps per gravity level per subject. GM Lf at 110° (bipedal standing on the platform) was 57.4 mm. GM at DO ranged from 46.5 mm (1 g, 130.4° ankle angle) to a minimum of 40.5 mm (137.1° ankle angle). *^a^p* < 0.05 1 g vs. 0–0.25 g; *^b^p* < 0.05 1 g vs. 0.25–0.5 g; *^c^p* < 0.05 1 g vs. 0.5–0.75 g; *^d^p* < 0.05 1 g vs. 0.75–1 g; *^dd^p* < 0.01 1 g vs. 0.75–1 g.

**Figure 9:**
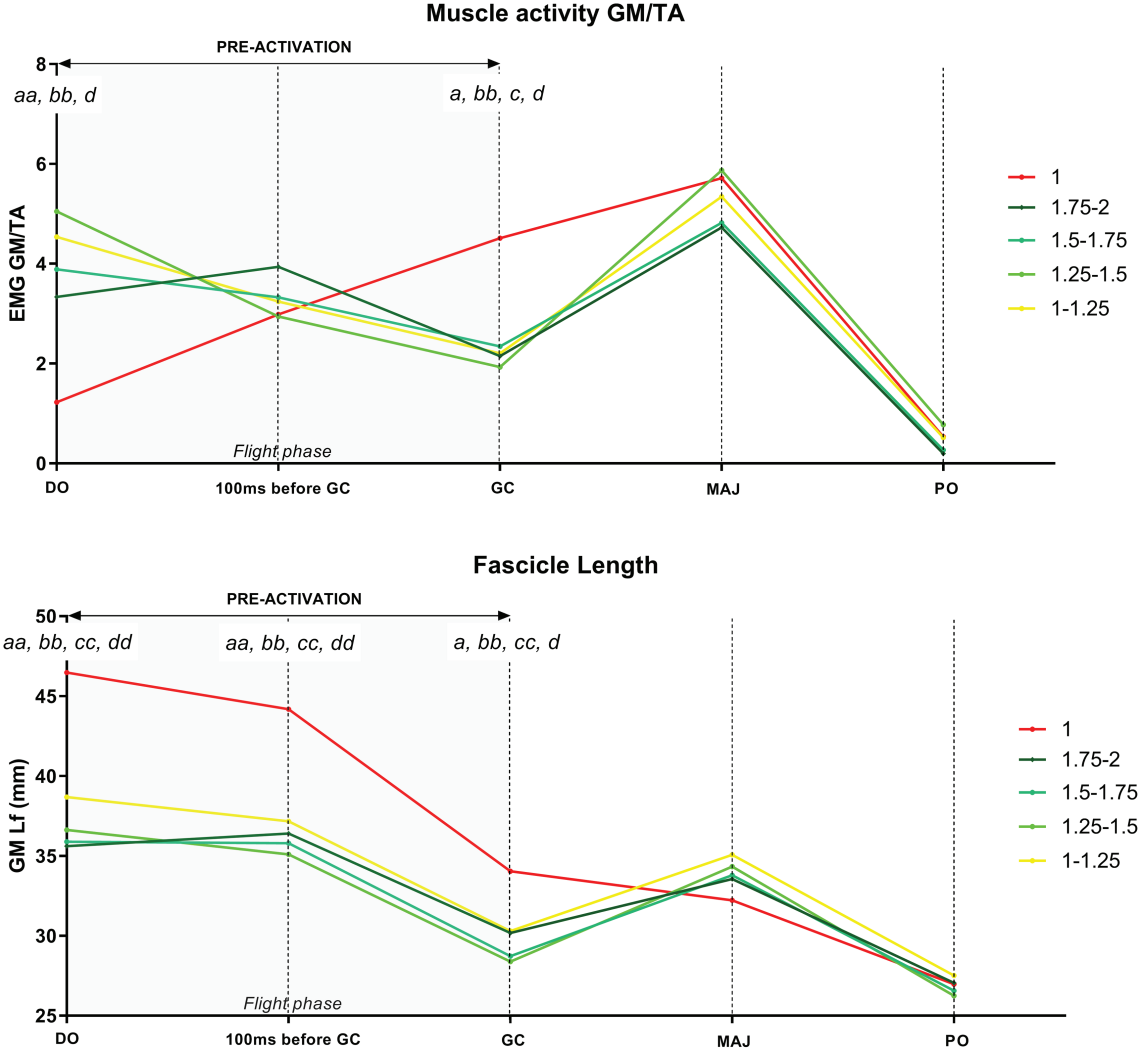
Time course of the gastrocnemius medialis/tibialis anterior (GM/TA) EMG and fascicle length (Lf) during the five drop jump time points – drop-off (DO), 100 ms before ground contact (GC), GC, MAJ, push-off (PO) – in hyper-gravity (1–1.25 g, 1.25–1.5 g, 1.5–1.75 g, and 1.75–2 g) and 1 g. Results shown as grand means of maximum three jumps per gravity level per subject. GM Lf at 110° (bipedal standing on the platform) was 57.4 mm. GM at DO ranged from 46.5 mm (1 g, 130.4° ankle angle) to a minimum of 35.6 mm (139.9° ankle angle). *^a^p* < 0.05 1 g vs. 1–1.25 g; *^aa^p* < 0.01 1 g vs. 1–1.25 g; *^bb^p* < 0.01 1 g vs. 1.25–1.5 g; *^c^p* < 0.05 1 g vs. 1.5–1.75 g; *^cc^p* < 0.01 1 g vs. 1.5–1.75 g; *^d^p* < 0.05 1 g vs. 1.75–2 g; *^dd^p* < 0.01 1 g vs. 1.75–2 g.

GM/TA EMG and Lf changes between hypo-gravity, hyper-gravity, and 1 g over the time course of DJs are shown in Figures 8, 9; mixed-effect analysis revealed significant jump time point, gravity, and jump time point × gravity interaction for both GM Lf and GM/TA EMG variables.

#### Drop-Off

During DO, GM Lf was shorter in all the hyper-gravity levels, but not the hypo-gravity ones, than in 1 g. The percentage difference ranged from 17.4% (1–1.25 g, *p* < 0.001) to 22.3% (1.5–1.75 g, *p* = 0.002).

While, as for Lf, no differences in GM/TA ratio were present between 1 g and hypo-gravity, this parameter was higher in hyper-gravity levels, pointing to a higher GM EMG activity (if compared to TA) in hyper-gravity already before starting the jump. GM/TA EMG difference between 1 g and hyper-gravity ranged from 9.2-folds in 1.75–2 g (*p* = 0.023) to 16-folds in the other clusters (1–1.25 g, *p* = 0.002; 1.25–1.5 g, *p* < 0.001; 1.5–1.75 g, *p* = 0.064).

**Figure 10:**
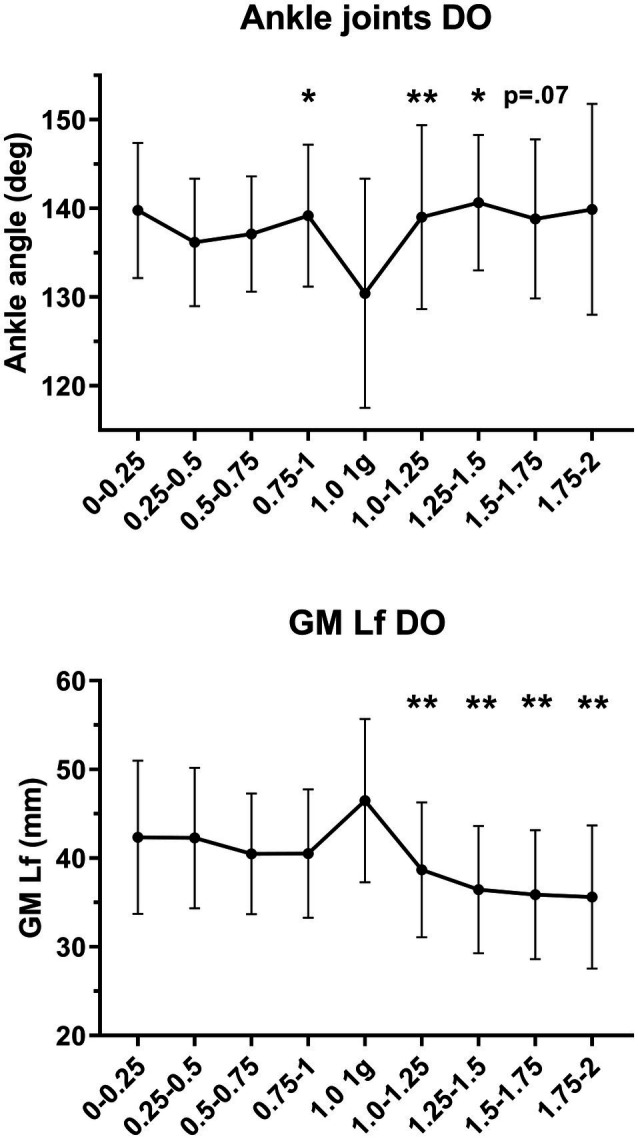
Ankle joint angles and gastrocnemius medialis (GM) fascicle length (Lf) at drop-off (DO) in all the hypo-gravity levels (0–0.25 g, 0.25–0.5 g, 0.5–0.75 g, and 0.75–1 g), hyper-gravity levels (1–1.25 g, 1.25–1.5 g, 1.5–1.75 g, and 1.75–2 g) and in 1 g. Results shown as mean ± SD of three jumps per gravity level per subject. ^*^p < 0.05 vs. 1 g; ^**^p < 0.01 vs. 1 g.

Ankle joint angle at DO in all the different gravity levels is shown in Figure 10.

At DO, ankle angle was 130.4° in 1 g.

Ankle joint angle was higher in hyper- and hypo-gravity if compared to 1 g; however, *post-hoc* analysis revealed that the difference was significant only in 0.75–1 g (139.2°, *p* = 0.162), 1–1.25 g (139.0°, *p* = 0.004), 1.25–1.5 g (140.7°, *p* = 0.010), and tended to be significant in 1.5–1.75 g (138.8°, *p* = 0.066).

**Figure 11:**
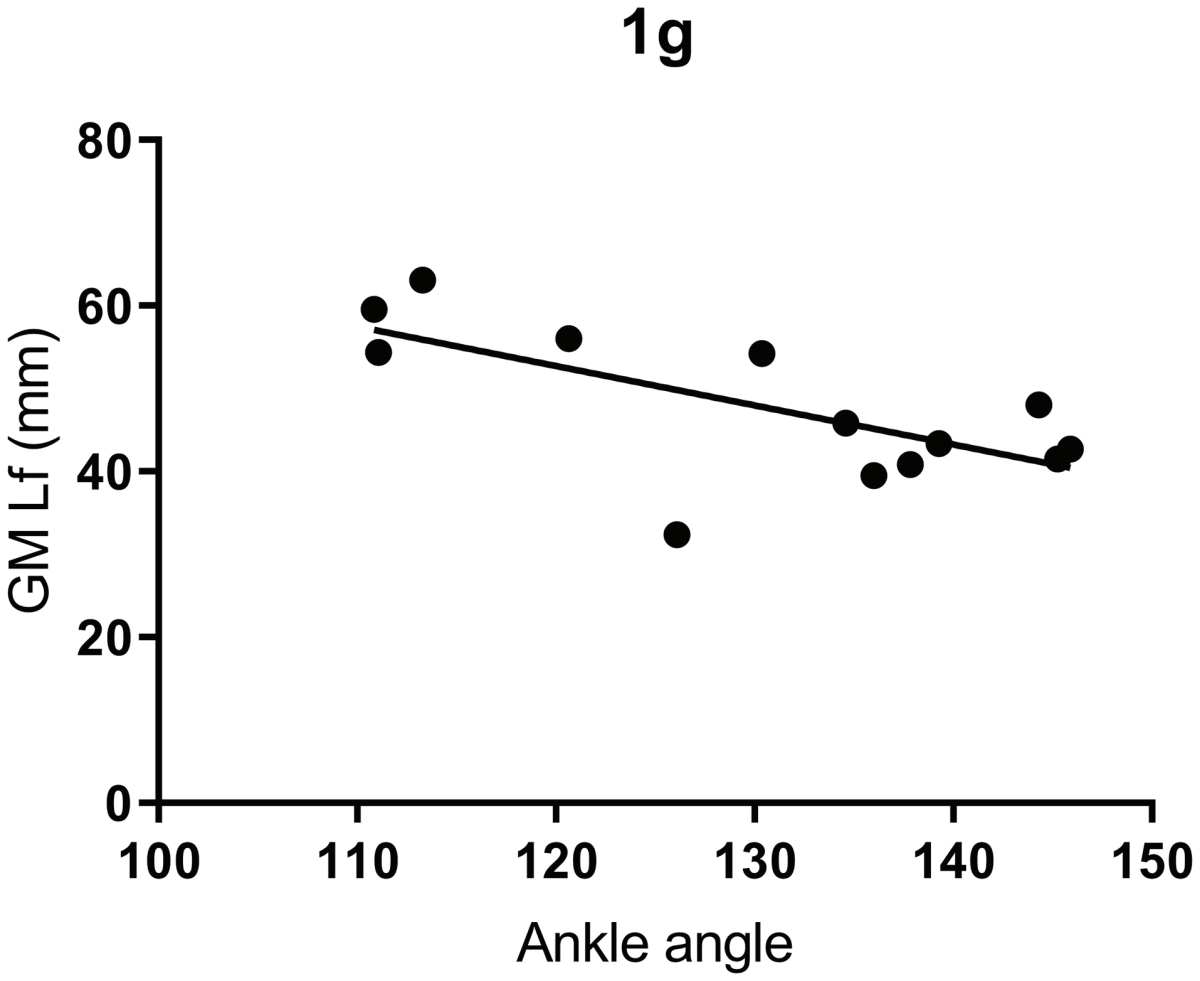
Correlation between gastrocnemius medialis (GM) fascicle length (Lf) and ankle joint angle at drop-off (DO) in 1 g. Correlation parameters: *p* = 0.0103, *r* = −0.68.

A significant correlation was found between ankle joint angle at DO and GM Lf at DO only in 1 g (*r* = −0.68; *p* = 0.010; Figure 11). The lack of correlation in all other gravity levels indicates that, below and above 1 g, ankle angle is not the major factor influencing GM Lf, highlighting the role of greater muscle activation as a possible contributor to the greater plantarflexion observed.

#### Pre-activation Phase

During the early pre-activation phase, at 100 ms before GC, Lf was significantly shorter in all the hyper-gravity levels and in 0.5–1 g compared to 1 g. Such difference ranged from 12.4% (0.5–0.75 g, *p* = 0.018) to 20.4% (1.25–1.5 g, *p* < 0.001). GM and TA EMG were similarly lowered in all the gravity levels if compared to 1 g, except for 0.25–0.5 g, where the ratio GM/TA EMG was significantly decreased (−41.1%, *p* = 0.017).

At the end of the pre-activation phase (GC), GM Lf was shorter only in hyper-gravity than in 1 g. The difference was by 11.0% (1–1.25 g, *p* = 0.011), 16.3% (1.25–1.5 g, *p* < 0.001), 15.7% (1.5–1.75 g, *p* = 0.008), and 9.8% (1.75–2 g, *p* = 0.050). Lf in 0.75–1 g showed a strong trend to be shorter than in 1 g (−7.7%, *p* = 0.058). GM EMG was significantly lower in all the gravity levels if compared to 1 g, with the exception of 1.75–2 g; in contrast, a higher co-activation of TA leads GM/TA ratio to be significantly lower in almost all the gravity levels, with the exception of 0.25–0.5 g (−34.8%, *p* = 0.064).

#### Braking Phase

At the end of the landing and braking phase (MAJ), Lf of GM was similar between 1 g and the majority of the other gravity levels, being significantly higher in 0–0.25 g (+19.4%, *p* = 0.045) compared to 1 g. GM and TA EMG were lower in all the gravity levels than in 1 g, once again to a similar extent; hence, GM/TA ratio showed no differences among gravities when compared to 1 g.

#### Push-Off Phase

At the PO moment, no differences in GM Lf and GM/TA EMG between 1 g and any other gravity level were observed.

### Variations of Sarcomere Length During DJ Along the Ascending Limb of the L-T Relationship

**Figure 12:**
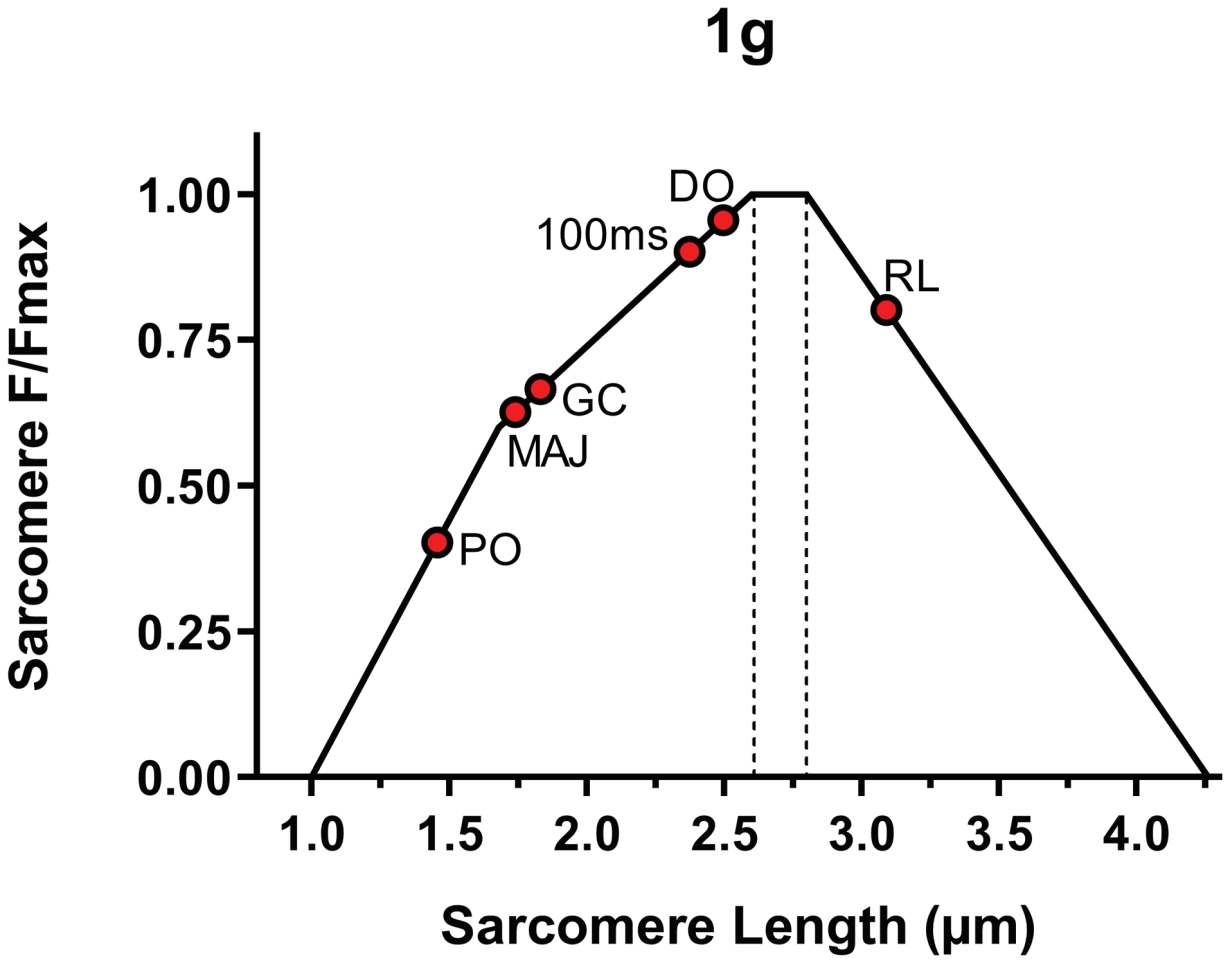
Operating sarcomere length in its length-tension (L-T) relationship during the five different drop jump time points in 1 g. L-T relationship graphs adapted from Walker and Schrodt (1974).

**Figure 13:**
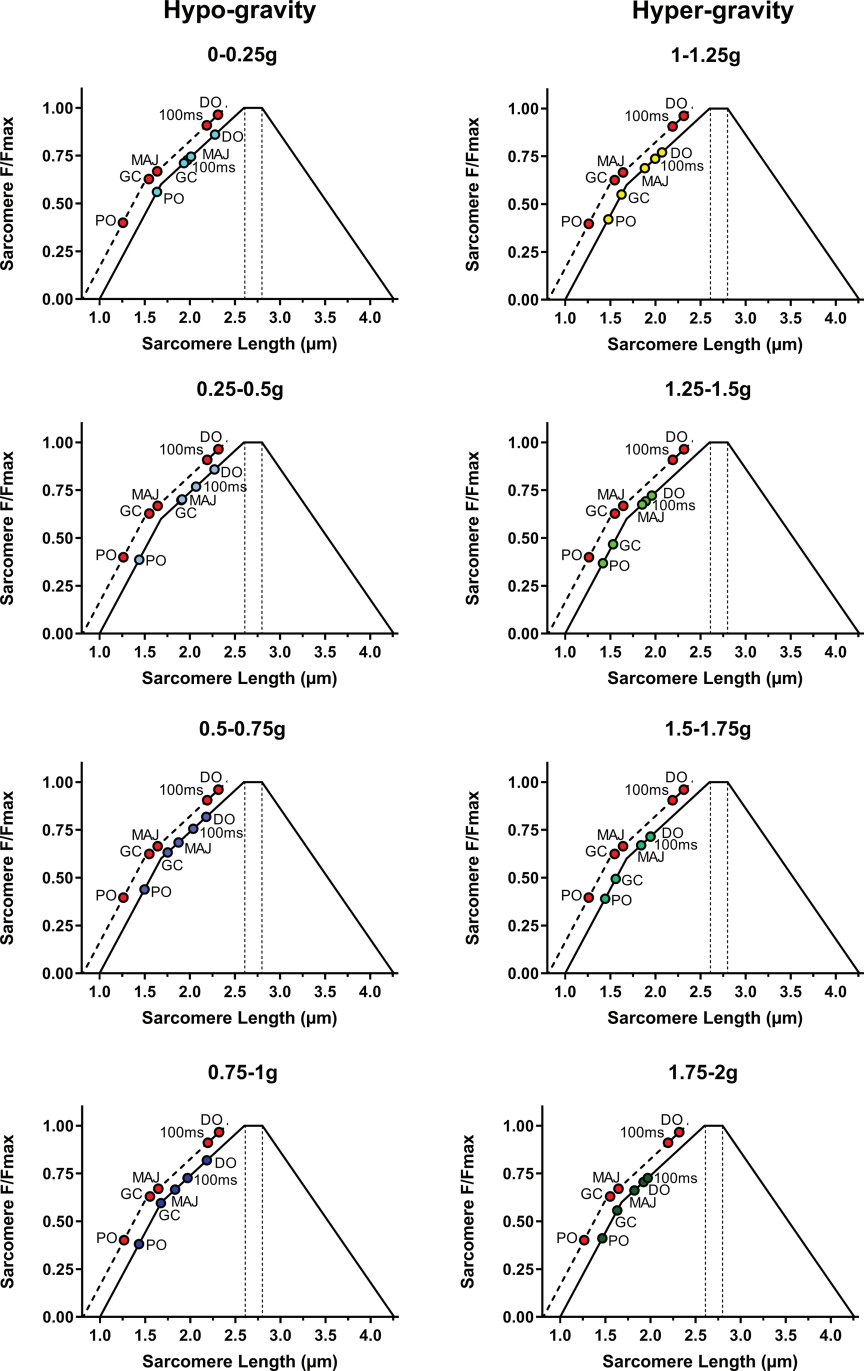
Operating sarcomere length in its length-tension (L-T) relationship during the five different drop jump time points in hypo-gravity and hyper-gravity. A comparison with the same drop jump time points in 1 g is also shown (dashed line, red dots).

The operating sarcomere length in the five different DJ time points for each gravity level is shown in Figures 12, 13.

At DO, the rotation of the joint in plantar flexion associated with a contraction (initial pre-activation) shifted sarcomere length (SL) from the standing value, theoretically located on the upper part of the descending limb of the L-T relationship [assumed to be 3.09 μm at 110° of joint angle (Sanchez et al., 2015)], to the upper part of the ascending limb of the L-T relationship. The shift was more pronounced in hypo-gravity (SL range: 2.18–2.28 μm) than in 1 g (SL = 2.50 μm) and even more marked in hyper-gravity (SL range 1.93–2.07 μm).

In 1 g, the contraction (pre-activation) and the plantar rotation, which continued during the flight phase, induced SL to further shorten until GC, reaching a value of 1.83 μm. In hypo-gravity levels, differences in SL between 0–0.5 and 0.5–1 g were observed: In the lowest gravities, SL at GC was higher (1.93–1.90 μm), whereas between 0.5 and 1 g, the SL was lower (1.75–1.67 μm) than in 1 g. In contrast, in hyper-gravity, SL at initial GC was always shorter (1.53–1.63 μm) than in 1 g.

The effect of landing on SL, as observed in GM Lf, differed depending on the gravity level. In 1 g, the strength of the contraction caused a further reduction in SL from 1.84 to 1.74 μm (phase GC-MAJ). In hypo-gravity, a very moderate sarcomere stretch was detectable, between 0.008 and 0.16 μm. In contrast, in hyper-gravity, muscle contraction appeared not sufficient to completely prevent the stretch which ranged between 0.19 and 0.32 μm. Such stretch brought SL back toward the plateau region, reaching values between 1.82 and 1.88 μm.

Overall, at MAJ, SL ranged between values that would allow to develop around the 70–80% of the maximum force in all the gravity levels ranging from 0–0.25 to 1.75–2 g.

In the last phase, from MAJ to PO, a strong contraction markedly reduced SL, which at the PO was rather similar in all gravities, ranging between 1.42 and 1.50 μm, with the only exception of 0–0.25 g, at which a value of 1.64 μm was reached.

Hence, during all the jump time points and at all the gravitational levels, GM sarcomeres seemed to be operating on the ascending limb of their L-T relationship.

## Discussion

This study was driven by two main research questions: (1) Is GM fascicle length differently regulated during DJs in variable gravity? and (2) How is muscle force production capability modified, based on estimated sarcomere length, in conditions of variable gravity?

In agreement with our first experimental hypothesis, we observed a gravity-dependent behavior of GM Lf at DO (the moment before starting the fall) and especially during the braking phase (GC-MAJ), where similar patterns in hypo- and hyper-gravity were recorded. However, at the end of the braking phase (MAJ), fascicles presented very similar lengths across all gravity levels, leading to similar Lf values at PO and thus optimizing fascicle mechanical behavior during the re-bounce phase. In line with fascicle behavior, sarcomere length also seemed to be differentially regulated, being leftward shifted in the ascending limb of the L-T relationship both in hypo- and hyper-gravity. An interesting finding was that, both in 1 g and in variable gravity, sarcomere operative length at PO was extremely short, ranging from 1.42 to 1.50 μm. This sarcomere length is far from the optimal one as it would allow to theoretically develop only 40% of sarcomere maximum force (Walker and Schrodt, 1974), implying a highly unfavorable condition for muscle mechanical output.

This intriguing result is discussed henceforward in terms of GM fascicle behavior in the different gravity levels.

### Fascicle Behavior and Estimated Sarcomere Operative Length in Normo-Gravity

#### Drop-Off

At DO, GM fascicles were on average 46.5 mm long at a joint angle of 130°, i.e., in slight ankle plantar flexion. When testing the existence of a relationship between individual GM Lf and ankle angle values (Figure 11), these were found to be significantly correlated, consistent with the findings of Narici et al. (1996). As expected, greater ankle angles (greater plantar flexion joint angles) were associated with shorter GM fascicles.

The corresponding estimated sarcomere length (2.50 μm) was close to the plateau of the L-T relationship (2.64 μm–2.81 μm) calculated by Walker and Schrodt (1974). This sarcomere length seems to be explained by the combination of ankle angle and muscle activity. Indeed, at 130°, GM sarcomere length determined during passive ankle movement (Narici et al., 1996) is expected to be 2.72 μm; thus, the observed shorter length is indicative of the contractile activity related to beginning of the pre-activation, which likely produces a further shortening of about 0.22 μm. The ability to develop force at this sarcomere length is very high, well above 90% of the maximum.

#### Pre-activation, Landing, and Push-Off

From DO, muscle fascicles followed a pattern very similar to that previously described during DJs (Fukashiro et al., 2006; Sousa et al., 2007): From DO to GC, a constant Lf shortening was observed; during the braking phase, fascicles firstly further shortened and consequently behaved quasi-isometrically, generating a high net impulse, of above 2,000 N, measured in terms of peak Z GRF difference between MAJ and GC (Figure 5); when the MAJ was reached (end of braking), a significant shortening of the fascicles was observed, accompanied by a marked drop in GM EMG activity (Bobbert et al., 1987; Ishikawa et al., 2005; Ritzmann et al., 2016).

According to the fascicle data from which they are estimated, from DO to GC, sarcomeres were found to shorten by about 27% (from 2.50 μm to 1.83 μm).

From GC to MAJ, sarcomere length remained almost constant, indicating that the muscle was operating quasi-isometrically. The relevance of this phase seems essential for forcing the tendon to elongate and store elastic energy, a result that is achieved by the muscle acting as an anchor for the elongating tendon. In addition, Fukashiro et al. (2005, 2006) emphasized that, relating to the muscle force-velocity curve, low contraction velocities (as the observed isometric contraction of the braking phase) would allow sarcomeres and, thus, fascicles, to produce higher forces to be transmitted to the tendon and, finally, bones, in the last PO phase. Similar observations were made by Fukunaga et al. (2001), investigating *in vivo* the behavior of GM fascicles and tendon during treadmill walking. These authors showed that PO is preceded by a quasi-isometric phase in which considerable tendon elongation occurs. Collectively, these observations emphasize the importance of the quasi-isometric phase preceding PO for enabling tendon elongation and storage of elastic energy.

What seems noteworthy is that at PO, extremely short sarcomere lengths, close to 1.5 μm, were reached. This finding may seem surprising, as at this sarcomere length, the contact between thick filaments and Z-lines is expected to drastically reduce the sarcomere force production capability, enabling to theoretically develop only about 40% of its maximum force. However, it is important to point out that this condition is reached with the ankle in full plantarflexion (since PO represents the moment before the ground is left to re-bounce), and most of the energy needed for this phase is provided by the release of elastic energy stored in the tendon from GC to MAJ and released from MAJ to PO. In fact, GM EMG can be seen to markedly decrease from MAJ to PO, confirming that tendon recoil is likely the major energetic contributor for PO. Moreover, interestingly, similar very short sarcomere operative lengths have been previously reported for GM fibers measured *in vivo* (Maganaris, 2003) and in cadavers (Cutts, 1988). Operative sarcomere lengths below 1.6 μm have also been described in muscles of the abdominal wall (Brown et al., 2012) and in the wrist flexors (Loren et al., 1996).

In summary, the present data suggest that in 1 g, the overall working range of the GM sarcomere from DO to PO is 2.50 to 1.46 μm, involving a change of sarcomere length of 42%. In different studies by Fukashiro and Kurokawa, concerning countermovement jumping and drop jumping in 1 g, the estimated working range of the GM sarcomere was calculated to be 3.4 to 2.0 μm and about 2.9 to 1.9 μm, respectively. Therefore, sarcomere shortening was estimated to be about 41 and 34% (Kurokawa et al., 2003; Fukashiro et al., 2005, 2006), quite similar to the value calculated in the present study. However, in these studies, the authors divided GM Lf (which was very similar to that found in our study) for a fixed number of sarcomeres (Bobbert et al., 1986). This could have led to a higher level of bias in the estimation of sarcomere length (thus obtaining higher values for operating sarcomere length at PO, close to 1.9–2 μm) when compared to the method used in this study, where individual GM Lf at rest was divided for the measured *in vivo* GM sarcomere length in the same position (Sanchez et al., 2015) to obtain an estimation of the number of sarcomeres in each participant muscle. Hence, although no definitive conclusions may be drawn from indirect estimations, it seems plausible that the sarcomere operative length reported in the present study may approximate the *in vivo* sarcomere behavior in the condition of normo-gravity (1 g).

### Fascicle Behavior and Estimated Sarcomere Operative Length in Variable Gravity

#### Drop-Off

When preparing to perform DJs in variable gravity (at DO), GM muscle fascicles and ankle joint behaved differentially in hypo- and hyper-gravity.

Indeed, hypo-gravity did not seem to significantly affect GM muscle behavior at this jump stage, as no differences in GM Lf and ankle joint angles were observable when comparing 1 g and the gravity levels below it. In contrast, GM fascicles were significantly shorter in hyper-gravity than in 1 g (Figure 9), and this was accompanied by a greater plantar-flexed joint angle and increased GM EMG and GM/TA EMG ratio. This might be foreseen as, due to the greater load on the postural muscles at DO, a higher excitatory drive of the motor cortex would be expected to increase the activation level before jump initiation (Post et al., 2009). GM EMG activity together with GM/TA EMG ratio increases, and the action of gravity itself, seem to potentially explain for the greater plantar-flexed joint angle and, thus, shorter Lf at DO in hyper-gravity. Sarcomere length, as inferred from fascicle length, was only slightly shorter in hypo-gravity than in 1 g, ranging between 2.18 and 2.27 μm, while being markedly shorter (1.93–2.07 μm) in hyper-gravity. Overall, in variable gravity, sarcomeres at DO appeared to be leftward shifted along the ascending limb of the L-T relationship when compared to the reference Earth condition.

#### Pre-activation

During pre-activation (from DO to GC), a reduction in GM activity was observed in all the jumps performed in variable gravity if compared to 1 g, and, in hyper-gravity, this was also accompanied by a slight increase in TA co-activation. In spite of this, similar or shorter GM Lf than 1 g was reached at the end of the pre-activation phase when jumping in gravity levels both below and above 1 g.

Although, at a fist sight, this may seem surprising, it should be considered that, in hypo-gravity, falling time is increased; it follows that the longer falling phase should enable fascicles and thus, sarcomeres, to shorten more in this condition compared to 1 g, despite the lower GM activity. The lower level of muscle activity may be advantageous in order to induce a reduction in reflex responses of the shank muscles (Ritzmann et al., 2016), leading to an increased compliance of the muscle, preferable when jumping in unknown conditions. Similarly, in hyper-gravity, a reduction in muscle stiffness may be preferred for safety reasons and the increased TA co-activation before landing likely occurred as a strategy to fix the joint angle in preparation for ground contact. Based on the experiments aimed at increasing the load through increases of DJ height (Santello and Mcdonagh, 1998; Ishikawa et al., 2005; Sousa et al., 2007; Lesinski et al., 2017) that showed increases in GM EMG with increasing fall height, one may expect a similar response when jumping in hyper-gravity. However, it must be considered that, differently from what happens with increasing fall height, jumping in hyper-gravity (1) leads to a reduction in falling time (thus reducing the pre-activation time) and (2) implies that the hyper-gravity forces are already acting on the body before the initiation of the drop-fall, as confirmed by the greater DO GM EMG and GM/TA ratio than in 1 g or hypo-gravity. Therefore, GM Lf is shorter at DO, needing less activation to reach the 10% shorter values observed at GC in hyper-gravity than in 1 g. A shorter fascicle length, and thus sarcomere length, may also be advantageous for enabling the fascicles to operate in the upper portion of the ascending limb of the L-T relation when landing in hyper-gravity, as higher loads upon ground contact have been shown to provoke GM fascicles lengthening (Sousa et al., 2007). Therefore, shorter fascicle lengths may allow the muscle to limit the lengthening contraction, reducing the risk of eccentric damage.

#### Landing and Push-Off

Upon landing (GC-MAJ), GM fascicles experienced a lengthening in the late braking phase in all the gravity levels below and above 1 g, although more markedly in hyper-gravity. This is in line with previous findings showing that, when jumping in unknown conditions (Helm et al., 2020), the quasi-isometric fascicle behavior observed when falling from optimal heights in normal gravity (Fukashiro et al., 2006) is lost. Such behavior is potentially due to the lower level of muscle activity in pre-activation and during landing (Waldvogel et al., 2021), which likely implies an increased muscle compliance and thus an increase in shock-absorption ability. Further, concerning hyper-gravity, several studies reported that falling from increasing heights (Ishikawa and Komi, 2004; Ishikawa et al., 2005; Sousa et al., 2007) leads to very similar results, i.e., the replacement of the quasi-isometric behavior of fascicles with a significant lengthening. Beside the increased muscle compliance, the higher loads acting on the muscle could cause greater fascicle lengthening due to the high gravity forces acting on it. According to fascicle data, contrary to 1 g in which little change in sarcomere length was found between GC and MAJ (quasi-isometric contraction), sarcomere length at MAJ was longer than at GC.

Interestingly, reflecting a similar pattern of GM fascicles and sarcomeres lengthening in hypo- and hyper-gravity during landing, the amount of force generated during this phase was significantly lower in both hypo- and hyper-gravity than in 1 g (Figure 5). Concerning hypo-gravity, this is consistent with previous findings also showing that the rate of force development upon landing is reduced (Ritzmann et al., 2016), likely due to the lower falling velocity and body weight, which implies that lower forces are necessary to generate a net impulse that allows to re-bounce in the PO phase. In contrast, in hyper-gravity, associated with fascicle lengthening, the contact time during landing is increased – thus reducing the explosiveness of the movement – and the muscular system has been shown to shift from an energy-storage to an energy-dissipation behavior (Waldvogel et al., 2021). Hence, the lower force generated might be the result of these elements, compromising DJ performance. Noteworthy, despite such decreased force production, the re-bounce at PO could be effectively performed, although the height of the vertical jump was reduced (Waldvogel et al., 2021).

Despite fascicle lengthening was observed during landing, it is noteworthy that, at the end of the braking phase, GM fascicles and sarcomeres reached lengths very similar to those in 1 g. This seems to suggest a fine regulation of GM and TA muscles throughout the whole jump, allowing Lf at MAJ to reach optimal values in order to perform a jump after landing. Similarly, at PO, very short GM Lf and sarcomere lengths were observed: Excluding 0–0.25 (sarcomere length = 1.63 μm), from 0.25–0.5 to 1.75–2 g, sarcomere lengths ranged between 1.42 and 1.50 μm, being close to 1 g estimated value (1.46 μm), suggesting that similar ankle angles would allow Lf and sarcomeres to reach lengths functional for movement performance.

### Methodological Considerations

When discussing the estimated sarcomere length, some critical issues must be considered:

1. Sarcomere length has been determined indirectly through a calculation which relies on direct measurements of fascicle length and on a published value of sarcomere length directly measured in standing position, which however is less reliable than directly measuring sarcomere length during the movement performance. The sarcomere length at each time point has been calculated from the ratio of fascicle length divided by the sarcomere number, the latter being obtained under the assumption that fiber length and fascicle length were identical. Anatomical observations (Monti et al., 1999) showed that fibers with different length can coexist and this would introduce uncertainty in the sarcomere number calculation. Recently, some techniques that allow the measurement of sarcomere length *in vivo* have been implemented. However, one of these, exploiting laser technology, has been only used in animal studies (Moo et al., 2016, 2017; Moo and Herzog, 2018), while the second, also applied on humans, consists of a portable tool that exploits a micro-endoscope with a needle inserted in the muscle (Llewellyn et al., 2008; Sanchez et al., 2015). This approach is presently suitable to determine sarcomere length in static conditions and not during movement. We have exploited for our calculation sarcomere length determined with this approach in gastrocnemius medialis, *in vivo*, in static condition, ankle angle = 110° (i.e., same condition as the one in which fascicle length was measured by ultrasound in our work).
2. Although widely employed, estimating theoretical sarcomere maximum force production capability from its length by using the L-T relationship proposed by Walker and Schrodt (1974) may not be completely correct. In fact, Walker and Schrodt L-T relationship was not experimentally obtained, as they used human actin length to calculate sarcomere length in Gordon’s model (Gordon et al., 1966), and human actin length has been shown to vary between different muscles (Gokhin et al., 2012). However, experiments by Ottenheijm et al. (2009) showed experimentally measured L-T relationships in human quadriceps femoris single fibers which were very similar to those calculated by Walker and Schrodt. In addition, the L-T relationship is obtained in conditions of isometric sarcomere length and maximal activation (Rassier et al., 1999): In our case, sarcomere length was not fixed (as DJ is a dynamic movement); however, we obtained five instantaneous frames (jump time points) of our fascicle and sarcomeres. In addition, we are aware that muscle activation was not maximal, which represents a further limitation of the proposed model.
3. In recent years, the non-uniformity of sarcomere length along and across the muscle belly has been reported (Moo et al., 2016, 2017; Moo and Herzog, 2018), and sarcomere length variability with muscle activation has been shown to be even more pronounced. In addition, it has now been well established that the force produced by muscles depend also on the absolute length and diameter of their fibers, which can span along the whole muscle length or taper within the connective tissue and experience a progressive decline in cross-sectional area along the length of the muscle. Thus, fiber absolute length and the difference in diameter across muscle region could determine different amounts of lateral force transmission, especially when sub-maximal contractions (only activating some motor units) occur (Heron and Richmond, 1993; Monti et al., 1999). These morphological features would inevitably affect the fiber and muscle L-T relationship *in vivo*, thus determining different force outputs. However, the aim of our estimations was to provide a general “average sarcomere operating length,” by using fascicle length during activity, as passive sarcomere length – possibly estimated from passive fascicle length – has been shown to be less reliable than active length to estimate force production capability (Moo et al., 2017). The rationale was therefore not to provide an estimation of the total force that might be produced by the muscle [which depends on different elements, many of them taken into account by the model proposed by Waldvogel et al. (2021)] but to isolate the average “sarcomere generating force capacity” during DJs in variable gravity.

Besides the above considerations, which imply that the real sarcomere length at PO might be non-uniform across the GM and possibly slightly higher or lower than the calculated one, it is important to stress the fact that GM fascicles were extremely short (between 26 and 28 mm) at PO. This implies that, since fascicle shortening is supposed to reflect sarcomere shortening [as it is reasonable to assume a correlation between the fascicle force-length relationship and the sarcomere force-length relationship (Fukashiro et al., 2005)], also sarcomeres are likely to operate at very short lengths during PO.

### Limitations

We acknowledge that this study has some limitations.

First of all, since parabolic flights could induce motion sickness, each volunteer participated as an operator to a flight before the one he was tested in. Although this procedure was necessary in order to avoid the possible insurgence of motion sickness to affect the results, participants were not completely naive to the exposure of hypo and hyper-gravity. Thus, we cannot exclude that the motor pattern observed, while jumping could have been partially influenced by the previous flight experience. Nonetheless, it is worth to note that, since volunteers performed DJs for 15 parabolas, no learning effect was observed within the parabolas, being the pattern very similar from the first to the last one; in addition, to avoid any confounding effect, we excluded parabola 1 and 2 from analysis.

Secondly, we are aware of the fact that during each parabola, the gravity force was continuously varying. Although the operator giving instruction to the participant had been previously trained and knew the correct time when the DJ had to be performed, gravity variations were very rapid, and therefore, jumps were not performed in the same exact gravity in each parabola and by each participant. However, to avoid drawing conclusion based on invalid results, we recorded, by using an accelerometer, the exact gravity during all the jump time points, for all the jumps and volunteers. Afterward, accelerometer data were used to cluster DJs in 9 different gravity clusters (see Methods): We believe that such strategy allowed us to draw reliable conclusions about the different muscle behavior within the different gravity levels.

## Conclusion

The present study shows that, when performing DJs in conditions of variable gravity, GM fascicles are differentially regulated in order to effectively perform the task required even in challenging conditions. Essentially, shorter GM Lf were observed at DO, allowing to reach, at MAJ and PO, similar lengths than in 1 g despite fascicle lengthening occurred during DJ braking phase in hypo- and, especially, hyper-gravity. Additionally, estimated sarcomere working range was found to be leftward shifted in the ascending limb of their L-T relationship in condition of variable gravity. However, it is noteworthy that a common value of sarcomere length was reached at PO, regardless of the gravity level, of about 1.5 μm for all conditions. This common value is probably dictated by the common ankle joint position at PO, the ankle being at right angle with the leg and the knee fully extended, allowing for the maximum possible tendon elongation and storage of elastic energy to be released in the final vertical jump.

## Data Availability Statement

The original contributions presented in the study are included in the article/supplementary material, further inquiries can be directed to the corresponding author.

## Ethics Statement

The studies involving human participants were reviewed and approved by Ethics Committee of the University of Freiburg (430/17). The patients/participants provided their written informed consent to participate in this study.

## Author Contributions

RR, AG, MN, JW, KF, MH, EM, and KA conceived and designed the research and performed the experiments. RR, JW, KF, EM, and CR analyzed the data. NC and PP provided new analytic tools to perform data analysis. RR, AG, MN, JW, KF, MH, EM, KA, and CR interpreted the results of the experiments, and edited and revised the manuscript. JW and EM prepared the figures. MN, EM, and CR drafted the manuscript. All the authors approved the final version of the manuscript.

## Conflict of Interest

The authors declare that the research was conducted in the absence of any commercial or financial relationships that could be construed as a potential conflict of interest.

## Publisher’s Note

All claims expressed in this article are solely those of the authors and do not necessarily represent those of their affiliated organizations, or those of the publisher, the editors and the reviewers. Any product that may be evaluated in this article, or claim that may be made by its manufacturer, is not guaranteed or endorsed by the publisher.

## Footnotes

^1^ http://imagej.nih.gov/ij/

## References

[ref1] Arampatzis A. Schade F. Walsh M. Brüggemann G.-P. (2001). Influence of leg stiffness and its effect on myodynamic jumping performance. J. Electromyogr. Kinesiol. 11, 355–364. 10.1016/S1050-6411(01)00009-8, PMID: 11595555

[ref2] Avela J. Santos P. M. Kyröläinen H. Komi P. V. (1994). Effects of different simulated gravity conditions on neuromuscular control in drop jump exercises. Aviat. Space Environ. Med. 65, 301–308. PMID: 8002909

[ref3] Bobbert M. F. Huijing P. A. van Ingen Schenau G. J. (1986). An estimation of power output and work done by the human triceps surae musle-tendon complex in jumping. J. Biomech. 19, 899–906. 10.1016/0021-9290(86)90185-5, PMID: 3793738

[ref4] Bobbert M. F. Huijing P. A. van Ingen Schenau G. J. (1987). Drop jumping II. The influence of dropping height on the biomechanics of drop jumping. Med. Sci. Sports Exerc. 19, 339–346. PMID: 3657482

[ref5] Brown S. H. M. Ward S. R. Cook M. S. Lieber R. L. (2012). Architectural analysis of human abdominal wall muscles: implications for mechanical function. Spine 36, 355–362. 10.1097/BRS.0b013e3181d12ed7, PMID: 21325932 PMC3017737

[ref6] Cutts A. (1988). The range of sarcomere lengths in the muscles of the human lower limb. J. Anat. 160, 79–88. PMID: 3253264 PMC1262051

[ref7] Fukashiro S. Hay D. C. Nagano A. (2006). Biomechanical behavior of muscle-tendon complex during dynamic human movements. J. Appl. Biomech. 22, 131–147. 10.1123/jab.22.2.131, PMID: 16871004

[ref8] Fukashiro S. Kurokawa S. Hay D. C. Nagano A. (2005). Comparison of muscle-tendon interaction of human m. gastrocnemius between ankle- and drop-jumping. Int. J. Sport Health Sci. 3, 253–263. 10.5432/ijshs.3.253

[ref9] Fukunaga T. Kubo K. Kawakami Y. Fukashiro S. Kanehisa H. Maganaris C. N. (2001). In vivo behaviour of human muscle tendon during walking. Proc. R. Soc. B Biol. Sci. 268, 229–233. 10.1098/rspb.2000.1361, PMID: 11217891 PMC1088596

[ref10] Gambelli C. N. Theisen D. Willems P. A. Schepens B. (2016). Human motor control of landing from a drop in simulated microgravity. J. Appl. Physiol. 121, 760–770. 10.1152/japplphysiol.00305.2016, PMID: 27516535

[ref11] Gokhin D. S. Kim N. E. Lewis S. A. Hoenecke H. R. D’Lima D. D. Fowler V. M. (2012). Thin-filament length correlates with fiber type in human skeletal muscle. Am. J. Physiol. Cell Physiol. 302, C555–C565. 10.1152/ajpcell.00299.2011, PMID: 22075691 PMC3287155

[ref12] Gollhofer A. Strojnik V. Rapp W. Schweizer L. (1992). Behaviour of triceps surae muscle-tendon complex in different jump conditions. Eur. J. Appl. Physiol. Occup. Physiol. 64, 283–291. 10.1007/BF00636213, PMID: 1592051

[ref13] Gordon A. M. Huxley A. F. Julian F. J. (1966). The variation in isometric tension with sarcomere length in vertebrate muscle fibres. J. Physiol. 184, 170–192. 10.1113/jphysiol.1966.sp007909, PMID: 5921536 PMC1357553

[ref14] Helm M. Freyler K. Waldvogel J. Lauber B. Gollhofer A. Ritzmann R. (2020). Anticipation of drop height affects neuromuscular control and muscle-tendon mechanics. Scand. J. Med. Sci. Sports 30, 46–63. 10.1111/sms.13550, PMID: 31487062

[ref15] Hermens H. J. Freriks B. Disselhorst-Klug C. Rau G. (2000). Development of recommendations for SEMG sensors and sensor placement procedures. J. Electromyogr. Kinesiol. 10, 361–374. 10.1016/S1050-6411(00)00027-4, PMID: 11018445

[ref16] Heron M. I. Richmond F. J. R. (1993). In-series fiber architecture in long human muscles. J. Morphol. 216, 35–45. 10.1002/jmor.1052160106, PMID: 8496969

[ref17] Ishikawa M. Komi P. V. (2004). Effects of different dropping intensities on fascicle and tendinous tissue behavior during stretch-shortening cycle exercise. J. Appl. Physiol. 96, 848–852. 10.1152/japplphysiol.00948.2003, PMID: 14594857

[ref18] Ishikawa M. Niemelä E. Komi P. V. (2005). Interaction between fascicle and tendinous tissues in short-contact stretch-shortening cycle exercise with varying eccentric intensities. J. Appl. Physiol. 99, 217–223. 10.1152/japplphysiol.01352.2004, PMID: 15705735

[ref19] Komi P. V. (1984). Physiological and biomechanical correlates of muscle function: effects of muscle structure and stretch-shortening cycle on force and speed. Exerc. Sport Sci. Rev. 12, 81–121. PMID: 6376140

[ref20] Komi P. V. (2000). Stretch-shortening cycle: a powerful model to study normal and fatigued muscle. J. Biomech. 33, 1197–1206. 10.1016/S0021-9290(00)00064-6, PMID: 10899328

[ref21] Koo T. K. Li M. Y. (2016). A guideline of selecting and reporting intraclass correlation coefficients for reliability research. J. Chiropr. Med. 15, 155–163. 10.1016/j.jcm.2016.02.012, PMID: 27330520 PMC4913118

[ref22] Kurokawa S. Fukunaga T. Nagano A. Fukashiro S. (2003). Interaction between fascicles and tendinous structures during counter movement jumping investigated in vivo. J. Appl. Physiol. 95, 2306–2314. 10.1152/japplphysiol.00219.2003, PMID: 12871964

[ref23] Lesinski M. Prieske O. Beurskens R. Behm D. G. Granacher U. (2017). Effects of drop height and surface instability on neuromuscular activation during drop jumps. Scand. J. Med. Sci. Sports 27, 1090–1098. 10.1111/sms.12732, PMID: 27460831

[ref24] Llewellyn M. E. Barretto R. P. J. Delp S. L. Schnitzer M. J. (2008). Minimally invasive high-speed imaging of sarcomere contractile dynamics in mice and humans. Nature 454, 784–788. 10.1038/nature07104, PMID: 18600262 PMC2826360

[ref25] Loren G. J. Shoemaker S. D. Burkholder T. J. Jacobson M. D. Fridén J. Lieber R. L. (1996). Human wrist motors: biomechanical design and application to tendon transfers. J. Biomech. 29, 331–342. 10.1016/0021-9290(95)00055-0, PMID: 8850639

[ref26] Maganaris C. N. (2003). Force-length characteristics of the in vivo human gastrocnemius muscle. Clin. Anat. 16, 215–223. 10.1002/ca.10064, PMID: 12673816

[ref27] Monti R. J. Roy R. R. Hodgson J. A. Reggie Edgerton V. (1999). Transmission of forces within mammalian skeletal muscles. J. Biomech. 32, 371–380. 10.1016/S0021-9290(98)00189-4, PMID: 10213027

[ref28] Moo E. K. Fortuna R. Sibole S. C. Abusara Z. Herzog W. (2016). In vivo sarcomere lengths and sarcomere elongations are not uniform across an intact muscle. Front. Physiol. 7:187. 10.3389/fphys.2016.00187, PMID: 27252660 PMC4879144

[ref29] Moo E. K. Herzog W. (2018). Single sarcomere contraction dynamics in a whole muscle. Sci. Rep. 8:15235. 10.1038/s41598-018-33658-7, PMID: 30323321 PMC6189036

[ref30] Moo E. K. Leonard T. R. Herzog W. (2017). In vivo sarcomere lengths become more non-uniform upon activation in intact whole muscle. Front. Physiol. 8:1015. 10.3389/fphys.2017.01015, PMID: 29270135 PMC5725405

[ref31] Narici M. V. Binzoni T. Hiltbrand E. Fasel J. Terrier F. Cerretelli P. (1996). In vivo human gastrocnemius architecture with changing joint angle at rest and during graded isometric contraction. J. Physiol. 496, 287–297. 10.1113/jphysiol.1996.sp021685, PMID: 8910216 PMC1160844

[ref32] Ottenheijm C. A. C. Witt C. C. Stienen G. J. Labeit S. Beggs A. H. Granzier H. (2009). Thin filament length dysregulation contributes to muscle weakness in nemaline myopathy patients with nebulin deficiency. Hum. Mol. Genet. 18, 2359–2369. 10.1093/hmg/ddp168, PMID: 19346529 PMC2694687

[ref33] Post M. Bakels R. Zijdewind I. (2009). Inadvertent contralateral activity during a sustained unilateral contraction reflects the direction of target movement. J. Neurosci. 29, 6353–6357. 10.1523/JNEUROSCI.0631-09.2009, PMID: 19439612 PMC6665515

[ref34] Rassier D. E. MacIntosh B. R. Herzog W. (1999). Length dependence of active force production in skeletal muscle. J. Appl. Physiol. 86, 1445–1457. 10.1152/jappl.1999.86.5.1445, PMID: 10233103

[ref35] Ritzmann R. Freyler K. Krause A. Gollhofer A. (2016). Bouncing on Mars and the Moon—the role of gravity on neuromuscular control: correlation of muscle activity and rate of force development. J. Appl. Physiol. 121, 1187–1195. 10.1152/japplphysiol.00692.2016, PMID: 27660301

[ref36] Roelants M. Verschueren S. M. P. Delecluse C. Levin O. Stijnen V. (2006). Whole body-vibration-induced increase in leg muscle activity during different squat exercises. J. Strength Cond. Res. 20, 124–129. 10.1519/R-16674.1, PMID: 16503671

[ref37] Sanchez G. N. Sinha S. Liske H. Chen X. Nguyen V. Delp S. L.. (2015). In vivo imaging of human sarcomere twitch dynamics in individual motor units. Neuron 88, 1109–1120. 10.1016/j.neuron.2015.11.022, PMID: 26687220 PMC5920519

[ref38] Santello M. Mcdonagh M. J. N. (1998). The control of timing and amplitude of EMG activity in landing movements in humans. Exp. Physiol. 83, 857–874. 10.1113/expphysiol.1998.sp004165, PMID: 9782194

[ref39] Shelhamer M. (2016). Parabolic flight as a spaceflight analog. J. Appl. Physiol. 120, 1442–1448. 10.1152/japplphysiol.01046.2015, PMID: 26796759

[ref40] Sousa F. Ishikawa M. Vilas-Boas J. P. Komi P. V. (2007). Intensity- and muscle-specific fascicle behavior during human drop jumps. J. Appl. Physiol. 102, 382–389. 10.1152/japplphysiol.00274.2006, PMID: 17068221

[ref41] Taube W. Leukel C. Gollhofer A. (2012). How neurons make us jump: the neural control of stretch-shortening cycle movements. Exerc. Sport Sci. Rev. 40, 106–115. 10.1097/JES.0b013e31824138da, PMID: 22089697

[ref42] Waldvogel J. Ritzmann R. Freyler K. Helm M. Monti E. Albracht K.. (2021). The anticipation of gravity in human ballistic movement. Front. Physiol. 12:614060. 10.3389/fphys.2021.614060, PMID: 33815134 PMC8010298

[ref43] Walker S. M. Schrodt G. R. (1974). I segment lengths and thin filament periods in skeletal muscle fibers of the Rhesus monkey and the human. Anat. Rec. 178, 63–81. 10.1002/ar.1091780107, PMID: 4202806

[ref44] Werkhausen A. Albracht K. Cronin N. J. Paulsen G. Bojsen-Møller J. Seynnes O. R. (2018). Effect of training-induced changes in achilles tendon stiffness on muscle-tendon behavior during landing. Front. Physiol. 9:794. 10.3389/fphys.2018.00794, PMID: 29997526 PMC6028711

[ref45] Wiley M. E. Damiano D. L. (1998). Lower extremity strength profiles in spastic cerebral plasy. Dev. Med. Child Neurol. 40, 100–107. 10.1111/j.1469-8749.1998.tb15369.x, PMID: 9489498

